# Transcriptome Analysis of Pterygium and Pinguecula Reveals Evidence of Genomic Instability Associated with Chronic Inflammation

**DOI:** 10.3390/ijms222112090

**Published:** 2021-11-08

**Authors:** María Fernanda Suarez, José Echenique, Juan Manuel López, Esteban Medina, Mariano Irós, Horacio M. Serra, M. Elizabeth Fini

**Affiliations:** 1 New England Eye Center, Tufts Medical Center and Department of Ophthalmology, Tufts University School of Medicine, Boston, MA 02111, USA; msuarez1@tuftsmedicalcenter.org; 2 Centro de Investigaciones en Bioquímica Clínica e Inmunología (CIBICI), Department of Clinical Biochemistry, Faculty of Chemistry, National University of Córdoba, Córdoba 5000, Argentina; jechenique@unc.edu.ar; 3 Instituto de Microcirugía Ocular Córdoba (IMOC), Córdoba 5000, Argentina; drlopezjuan@gmail.com (J.M.L.); estebanhmedina@gmail.com (E.M.); ma_iros@hotmail.com (M.I.); 4 Programs in Cell, Molecular & Developmental Biology and Pharmacology & Drug Development, Tufts Graduate School of Biomedical Sciences, Boston, MA 02111, USA

**Keywords:** ocular surface epithelia, conjunctiva, pinguecula, pterygium, genomic instability, inflammation, immune response, cancer

## Abstract

Solar damage due to ultraviolet radiation (UVR) is implicated in the development of two proliferative lesions of the ocular surface: pterygium and pinguecula. Pterygium and pinguecula specimens were collected, along with adjacent healthy conjunctiva specimens. RNA was extracted and sequenced. Pairwise comparisons were made of differentially expressed genes (DEGs). Computational methods were used for analysis. Transcripts from 18,630 genes were identified. Comparison of two subgroups of pterygium specimens uncovered evidence of genomic instability associated with inflammation and the immune response; these changes were also observed in pinguecula, but to a lesser extent. Among the top DEGs were four genes encoding tumor suppressors that were downregulated in pterygium: *C10orf90*, *RARRES1*, *DMBT1* and *SCGB3A1*; *C10orf90* and *RARRES1* were also downregulated in pinguecula. Ingenuity Pathway Analysis overwhelmingly linked DEGs to cancer for both lesions; however, both lesions are clearly still benign, as evidenced by the expression of other genes indicating their well-differentiated and non-invasive character. Pathways for epithelial cell proliferation were identified that distinguish the two lesions, as well as genes encoding specific pathway components. Upregulated DEGs common to both lesions, including *KRT9* and *TRPV3*, provide a further insight into pathophysiology. Our findings suggest that pterygium and pinguecula, while benign lesions, are both on the pathological pathway towards neoplastic transformation.

## 1. Introduction

Although the eye depends on the energy from visible radiation to carry out its fundamental physiological processes, it can also be damaged by this energy, as well as by ultraviolet radiation (UVR). Ophthalmoheliosis disorders are eye diseases in which sunlight is implicated, and these conditions are significant eye health hazards in many communities worldwide [1]. Examples of ophthalmohelioses that occur at the ocular surface are pterygium [2] and pinguecula [3].

The term “pterygium” is a Latinized version of the Greek word “pterygion” meaning “small wing”. Pterygium comprises a wing-shaped fibrovascular tissue that grows progressively from the corneoscleral limbus to the center of the cornea. This can lead to visual impairment, astigmatism, and dry eye [4]. A fully developed pterygium presents a well-formed head (apical aspect present on the cornea), a body (conjunctival aspect extending between the limbus and the canthus), and a neck (limbal aspect) [5]. Pterygium can be classified morphologically as atrophic, intermediate or fleshy, and histologically as angiomatous, fibrous or mixed [6]. The epithelium is normal, thinned, hyperkeratotic or hyperplastic, and the stroma exhibits nodular elastotic degeneration. Surgical excision is the only effective treatment, but recurrence is frequent.

The term “pinguecula” originates from the Latin word “pinguis,” which means grease. It is a well-defined nodular lesion that occurs near the corneal limbus in the interpalpebral fissure. Although it does not affect vision, it may cause cosmetic complaints and surgical excision may be considered for cosmetic reasons. Pinguecula is much like pterygium, except that growth of the lesion does not cross into the cornea. Pinguecula lacks vascularization in grade 0 and grade 1 [7], but grade 2 is highly vascularized and presents as an elevated lesion [8].

Ophthalmoheliosis disorders occur more commonly in people that reside close to equatorial latitudes, where UVR intensity is highest. The so-called “pterygium belt” [9] lies between 37° north and south of the equator. Pterygium and pinguecula occur at greater incidence in older individuals and commonly cited risk factors include time spent outdoors and exposure to wind and dust [10,11,12]. A wide range of other risk factors has been proposed for pterygium, including genetic predisposition, viral infection, epigenetic aberration, immunologic disorder, inflammation and dysregulation of lipid metabolism [13,14,15]. Hard contact lens wear has been proposed as a risk factor for pinguecula [16].

Despite many years of study, the underlying molecular mechanisms leading to the development of pterygium and pinguecula are still relatively unknown. Gene expression profiling is an unbiased approach which can provide clues for hypothesis development. Several studies have used gene expression microarray platforms or profiling of expressed sequence tags to compare differentially expressed genes (DEGs) in pterygium and healthy conjunctiva [17,18,19,20,21,22,23]. Very recently, RNA sequencing (RNA-seq) technology was applied for the first time [24,25].

RNA-seq provides the opportunity to quantitatively and comprehensively define the transcriptomes of diseased tissues, and to identify DEGs differentiating healthy tissues from disease lesions. Such data can be used to develop a molecular profile of the disease lesions and further analysis using computational analysis tools can provide an insight into pathogenesis. Here we used RNA-seq to compare the transcriptomes of pterygium and pinguecula to the healthy conjunctiva, and to each other.

## 2. Results

### 2.1. DEG Profiles

Histologic findings in pterygium typically include a migratory epithelial front with proliferative features, epithelial atypia and dysplasia, epithelial squamous metaplasia, hyperplasia of goblet cells, increased pigmentation, disrupted underlying Bowman’s layer, fibrovascular proliferation, elastotic extracellular matrix (ECM) and inflammatory infiltration. However, individual specimens may lack some of these characteristics or even present with opposite features. To provide a reference for variability of pathological characteristics of pterygium to compare to our RNA-seq findings, we chose 3 studies from the literature with large case series, one of which included both primary and recurrent pterygia cases. Comparative characteristics are listed in Supplementary Table S1. The 3 studies took place in different parts of the world, both inside (Sydney and Cairo) [26,27] and north of the pterygium belt (Montreal) [28]. In the current study, specimens were collected from individuals residing near Córdoba, Argentina, south of the pterygium belt.

Next we sequenced the RNA extracted from specimens of normal healthy conjunctiva, pinguecula, and pterygium. The pterygium specimens were split into two subgroups based on whether they were derived from individuals that worked primarily outdoors or primarily indoors, thus experiencing different degrees of UVR exposure. The two subgroups were named pterygium highly exposed (pterygium-E) and pterygium normally exposed (pterygium-NE). We identified transcripts from 18,630 different genes in the normal conjunctival samples. Their expression levels were compared to expression levels in pinguecula and pterygium specimens. Pairwise comparisons were made among the groups, with the DEG criterion set as a change ≥1.5-fold. The number of DEGs across the pairwise comparisons is depicted in Table 1. Pinguecula and pterygium exhibited 27–34% similarity to normal health conjunctiva. Pinguecula and pterygium exhibited 95% similarity to each other. Pterygium-E and pterygium-NE exhibited 99.2% similarity to each other.

### 2.2. Validation of RNA-Seq Data

**Figure 1:**
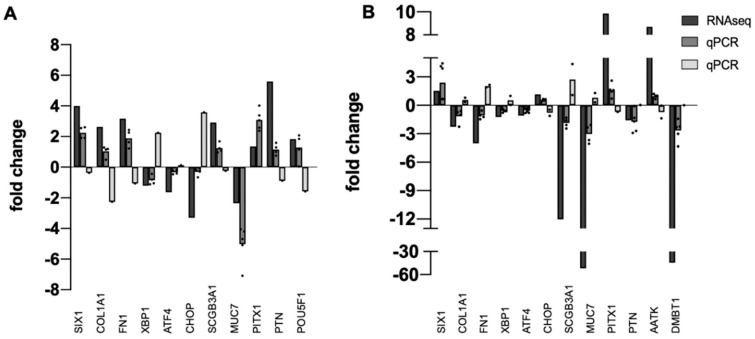
Validation of RNA-seq Data. Bar graph showing the relative correlation between RNA-seq data and qPCR data. (**A**) pinguecula vs. conjunctiva. (**B**) pterygium-E vs. conjunctiva. Black bars indicate fold change of gene transcripts in RNA-seq data; gray bars indicate fold change of gene transcripts determined by qPCR (dark gray = downregulated; light gray = upregulated). A normalized ratio (Y-axis) of more than 1 indicates upregulation, whereas a ratio of less than one indicates downregulation. The X-axis shows a random selection of 13 genes, *n* = 4 or 5 for each determination, with dot plots used to display the range of data points.

Next, independent tissue specimens were collected for validation of RNA-seq data. Thirteen upregulated or downregulated DEGs identified by RNA-seq were randomly selected and qPCR was used to quantify fold-change in pterygium or pinguecula vs. healthy conjunctiva in the new specimens. Results are shown in Figure 1. All DEGs from the RNA-seq analysis were also DEGs in the qPCR analysis. Direction of change (plus or minus) was mostly consistent with RNA-seq data, with a few exceptions, expected because of the known variability of pathological phenotypes (Supplementary Table S1).

### 2.3. Top DEGs

We identified top upregulated and downregulated DEGs in pinguecula, pterygium-E and pterygium-NE by aligning the gene lists for each analysis in adjacent columns in Microsoft Excel (Microsoft Corp., Redmond, WA, USA), and then sorted them by the column for each tissue using the Excel sorting tool. We then researched the top DEG function by accessing information in GeneCards Suite (Weizmann Institute of Life Science, Rehovot, Israel), and by literature searches on PubMed (National Institutes of Health (NIH), National Library of Medicine (NLM), National Center for Biotechnology Information (NCBI)). As an assessment of reproducibility, we compared our findings to those of the previously published gene expression profiling studies noted in the Introduction to this paper [18,19,20,22,24,25], as well as to studies that investigated expression of individual genes (discussed more below).

### 2.4. Top 25 Upregulated DEGs

The top 25 upregulated DEGs are listed in Table 2. Gene product functions are detailed in Supplementary Table S2. Expression of all top upregulated DEGs was changed > 5-fold with respect to conjunctiva. Most were upregulated in both pterygium subgroups, with only the amount of change being different. Thus, of the top 25 upregulated DEGs in pterygium-E, 21 were also upregulated in pterygium-NE (84% similarity). Of the top 25 upregulated DEGs in pterygium-NE, 22 were also upregulated in pterygium-E (88% similarity).

Pinguecula exhibited less, but still a substantial similarity. Of the top 25 upregulated DEGs in pterygium-E, 14 were also upregulated in pinguecula (56% similarity). Of the top 25 upregulated DEGs in pterygium-NE, 13 were also upregulated in pinguecula (52% similarity). In apparent contradiction, only 8 of the top 25 upregulated DEGs in pinguecula were also upregulated in pterygium-E, and only 4 in pterygium-NE. However, most of this difference can be explained by the relative levels of lymphocyte infiltration. Leukocyte infiltration is a hallmark of both pterygium and pinguecula, although the number of infiltrating cells and the profiles of cell types is quite variable (see Supplementary Table S1). Thirteen of the top pinguecula DEGs are expressed by lymphocytes and implicated in their biology: *CD2*, *CCR2*, *IDO1*, *IGHA1*, *IGHA2*, *IGHG1*, *IGHM*, *IGKC*, *IGLC2*, *IGLC3*, *LGR5*, *NR2F1*, *SFRP2*. Seven of these genes encode immunoglobulin (IgG) chains, which are associated primarily with lymphocytes of the B lineage [29].

Tumor cells acquire and metabolize glucose at high rates, allowing for the shunting of glycolytic intermediates toward biosynthetic pathways to meet proliferative demands [30]. Two of the top upregulated DEGs—*LDHAL6B* and *PCK1*—encode metabolic enzymes of the glycolysis/gluconeogenesis pathway. *TMPRSS11B* encodes a transmembrane proteinase which was also upregulated in squamous cell carcinoma of the lung. It promotes tumor growth by enhancing lactate export [31]. Two additional top upregulated DEGs—*ALDH1* and *ADH1C*—encode alcohol dehydrogenase family members that might also act to enhance this pathway [32]. *PCK1* and *ALDH* genes are also markers for corneal limbal stem cells, and their gene products regulate cellular functions related to self-renewal, expansion, differentiation, and resistance to drugs and radiation [33,34].

Two upregulated DEGs may provide protection against UVR exposure. *LGI3* expression has not been reported previously in ocular surface epithelia, but is highly expressed in the epidermis of skin [35]. UVR stimulates the secretion of *LGI3* protein, which protects against deleterious effects [35]. *NPIPA3* encodes a nuclear pore complex-interacting protein. The one publication we found on this protein reports on its localization to retinal rod photoreceptors, with the highest expression in the macula where light is focused for sight [36].

These DEGs are considered more in the next sections:

- DNA damage response: *AATK*
- Inflammation and immune response: *CLEC18A*, *FOSL1*, *PPMN1*
- Epithelial cell proliferation: *IGF1*
- Epithelial cell differentiation: *KRT9*
- Epithelial cell fate: *PITX1*, *POU5F1*, *SNAI1*
- Epithelial cornification: *SPRR3*, *IVL*, *CAPN14*
- Mucosal differentiation: *MUC6*
- Fibrovascular proliferation: *HBA1*, *PI16*, *POSTN*, *RP1-261G23.7*, *SLC26A4-AS1*

### 2.5. Top 25 Downregulated DEGs

The top 25 downregulated DEGs are listed in Table 3. Functions of their gene products are detailed in Supplementary Table S3. Expression of all DEGs was changed > 5-fold with respect to conjunctiva. Essentially, all were downregulated in both pterygium subgroups, with only the amount of change being different. Pinguecula exhibited a lesser, but still substantial similarity. Thus, of the top 25 DEGs downregulated in pterygium-E, 16 were also downregulated in pinguecula (64% similarity). Of the top 25 DEGs downregulated in pterygium-NE, 19 were also downregulated in pinguecula (76% similarity). Of the top 25 DEGs downregulated in pinguecula, 20 were also downregulated in pterygium-E (80% similarity), and 19 were also downregulated in pterygium-NE (76% similarity).

Most interesting were the 4 genes that function as tumor suppressors: *C10orf90*, *RARRES1*, *DMBT1*, and *SCGB3A1*. All 4 genes were downregulated in pterygium-E and pterygium-NE. *C10orf90* and *RARRES1* were also downregulated in pinguecula.

*C10orf90* (Fragile Site Associated Tumor Suppressor; FATS) encodes an intracellular protein that promotes the activation of TP53 in response to DNA damage, leading to a robust checkpoint response [37]. The gene is located on the chromosome at a specific region of genomic instability known as a common fragile site, which is susceptible to deletion in tumors induced by ionizing radiation.

*RARRES1* (Retinoic Acid Receptor Responder 1) encodes a type I membrane protein. When *RARRES1* is depleted from epithelial cells, they rewire the glucose metabolism by switching it from aerobic glycolysis to glucose-dependent de novo lipogenesis [38]. This is consistent with our observation of glycolysis/gluconeogenesis gene upregulation (Table 2). An early microarray study also reported downregulation of *RARRES1* in pterygium [18]. In addition, the previous RNA-seq study reported *RARRES1* as one of the top downregulated DEGs in pterygium [24].

*DMBT1* (Deleted in Malignant Brain Tumors 1) and *SCGB3A1* (Secretoglobin Family 3A Member 1) encode small secreted proteins expressed at high levels by mucosal tissues throughout the body [39,40,41]. An early microarray study also reported downregulation of *SCGB2A1* in pterygium [18]. Moreover, the previous RNA-seq study reported *SCGB3A1* as one of the top downregulated DEGs in pterygium [24]. However, we observed some variability in expression of this gene in our validation study, with some specimens showing upregulation (Figure 1).

Six of the top downregulated DEGs of this study encode proteins involved in the immune response. *IGHG3* encodes an IgG heavy chain that is downregulated in pterygium, but is upregulated in pinguecula (like other IgG encoding genes listed in Table 2). *FCGR3A* encodes an Fc receptor for IgG and *FAIM3* encodes an Fc receptor for IgM. *CCL18* is a chemokine that attracts T-cells into tissues. *C3* encodes complement factor 3 and *MRC1* encodes a mannose receptor of the lectin pathway for complement activation.

Curiously, several genes encoding molecular chaperones of the heat shock protein HSP70 family—*HSPA1A*, *HSPA1B*, *HSPA6*, *HSPA7*—were among the top downregulated DEGs in pinguecula. When we queried our complete datasets, we found a total of 27 HSP family members were expressed and 14 were downregulated in pinguecula (~50%). However, expression of these genes was unchanged or modestly upregulated in pterygium-E, and were unchanged in pterygium-NE. We considered the idea that heat shock gene downregulation in our pinguecula specimens might be related to the high upregulation of IgG gene expression we observed in pinguecula. Searching the literature, we found one study reporting the downregulation of *HSPA1A* in tumor cells by intravenous administration of IgG, due to high titers of anti-HSPA1A antibodies [42].

These DEGs are considered more in the next sections:

- Mucosal differentiation: *MUC7*
- Fibrovascular proliferation: *HBA1*, *HBA2*, *HBB*

### 2.6. Top Diseases and Biological Functions

To determine how the DEGs identified in our tissue comparisons might contribute to pathology, we used the Ingenuity Pathway Analysis (IPA) software from Qiagen (Redwood, CA, USA). After loading our datasets, we applied the IPA Top Diseases and Bio Functions tool, which groups genes based on their direction of change. As shown in Table 4, the disease or biological function with the top *p*-value was “Cancer, Organismal Injuries and Abnormalities” for all 6 tissue comparisons. In these comparisons, different cancer types within the general category were identified as activated or inhibited by their z-score. This finding emphasizes the often-noted similarities between pterygium and cancer [43].

Because the small number of DEGs in this comparison made it feasible, we researched them individually in IPA, GeneCards Suite and PubMed. Results of our analyses are summarized in Table 5, and are ranked by the change of expression in pterygium-NE.

We identified 13 DEGs that comprise a category we named “DNA Damage Response, Mitotic Checkpoints and Cell Cycle”. This includes 5 genes in the IPA subcategory of “Cell Cycle”, as well as 8 others differentially regulated in pterygium-E vs. pterygium-NE. It was previously reported that genes involved in double-strand DNA break repair, *RAD50*, *RAD51*, *XRCC2* and *XRCC3*, are differentially expressed in pterygium [44]. In addition, *PLK1*, which regulates the activity of RAD51, was identified as highly expressed in pterygium [45]. We already had *XRCC2* listed; we added *RAD50*, *RAD51*, *RAD51B*, *RAD51C*, *RAD51D*, and *XRCC3*. Finally, we added three genes from the top upregulated list involved in the DNA damage response: *AATK*, *PLD6*, *SLC26A4-AS1*, for a total of 25 genes. Genes are listed in order of expression level in pterygium-NE (lowest to highest). Expression of 17/25 of these genes was changed in pterygium-NE, but only 12/25 in pterygium-E. Expression of only 7/25 was changed in pinguecula. Moreover, many of the expression changes were in opposite direction in pterygium-E vs. pterygium-NE.

These interesting findings suggest genomic instability in pterygium, and in pinguecula at a lower level. The variability between pterygium samples is consistent with the variability of epithelial atypia and dysplasia observed in pterygium (Supplementary Table S1).

*TP53* encodes a transcription factor which is normally rapidly degraded. However, once stabilized and activated via posttranslational modifications, it protects damaged cells against malignant transformation by inducing cell cycle arrest, senescence, or death. In normal somatic cells, TP53 protein levels increase in response to DNA damage [46]. The protein accumulates in all layers of the corneal and conjunctival epithelium [47]. Expression of *TP53* did not change in pterygium or pinguecula. However, expression of TP53 downstream target *CDKA1A*, which encodes cell cycle inhibitor p21, increased in pterygium. Consistent with this, *MKI67*, which encodes a marker of cell proliferation, is downregulated in pterygium. Reduced cell proliferation in our pterygium specimens, as compared to healthy conjunctiva, suggests an epithelial atrophy morphology (Supplementary Table S1).

Genes in the IPA subcategories “Organismal Injury, Inflammatory Response”, “Dermatological Diseases and Conditions”, and “Immunologic Disease” overlap considerably, with a total of 24 unique genes. We grouped them together under a new category that we named “Inflammation and Immune Response”. We added 4 of the top upregulated genes in pterygium: *CLEC18A*, *FOSL1*, *PPMN1* and *SPRR3*. Expression of ~half of these genes changed in pinguecula, with 10/14 downregulated. Even more of these genes changed in pterygium, many in opposite direction when comparing pterygium-E vs. pterygium-NE.

### 2.7. Cell Type Signatures

A recent study used single cell RNA sequencing (scRNA-seq) to characterize cell types in the normal human cornea and conjunctiva [48]. Table 6 lists the twenty gene expression clusters identified in that study, showing the differential expression patterns of their signature genes in pterygium and pinguecula.

Signatures for most of the epithelial cell types identified in the previous study were represented in our tissues. This is consistent with results of a previous immunolocalization study reporting a mixed population of corneal-, limbal- and conjunctival-like cells in pterygium [22].

Relative marker expression levels suggest more suprabasal squamous epithelial cell layers in pterygium-NE as compared to pterygium-E, a more pigmented epithelial layer in pinguecula as compared to pterygium, and a more fibrovascular stromal layer in pinguecula as compared to pterygium. These differences are within the range of phenotypic variability for pterygium and pinguecula (Supplementary Table S1).

Signature genes for the two epithelial limbal progenitor cell types (cluster 10, cluster 9) that express *TP63* were unchanged or downregulated in both pterygium and pinguecula. These may correspond to the small, non-proliferating TP63-positive cells called “Fuchs’ flecks”, previously identified in association with corneal-like epithelial cells at the pterygium head [26]. Significantly, expression of marker genes for the quiescent stem cell types of both the epithelium (cluster 13) and stroma (cluster 3) was not detectable.

Stromal keratocyte and fibroblast signatures were also detected, but not the corneal endothelial cell signature. Epithelial cell type marker genes were relatively more upregulated in our pterygium specimens, while stroma cell type markers were more downregulated; the reverse was observed for pinguecula. Expression of signature genes for cells distinguishing blood vessels, limbal vessels and melanocytes were upregulated in pinguecula, but downregulated in pterygium.

Our evaluation of cell type signatures defined in a study in mouse [49] led to similar conclusions (Supplementary Table S4).

### 2.8. Upstream Regulators

To determine signaling pathways that might be activated or inhibited in pterygium and/or pinguecula, we used the upstream regulator tool in IPA, which predicts potential regulators based on the direction of change of DEGs. Table 7 lists upstream regulators that the IPA predicts to activate or inhibit their pathways in pterygium, grouped by functional categories.

#### 2.8.1. Epithelial Cell Proliferation

The largest category that we created is “Epithelial Cell Proliferation”, with 12 different upstream regulators that IPA predicts to regulate activated pathways, as shown in Table 7. Supplementary Table S5 compares expression of genes in these pathways across pterygium and pinguecula.

It is known that two major growth-factor signaling cascades regulate epithelial cell proliferation. One is activated by IGF1 or high insulin. *IGF1* is one of the top upregulated genes in pinguecula, and is produced by corneal epithelial cells [50]. It binds to cell surface receptor IGF1R, leading to activation of the PI3K-AKT pathway and the RAS-MAPK pathway. Ternary complex formation between IGF1R and integrins ITGAV: ITGB3 or ITGA6:ITGB4 are essential for signal transduction. The IPA upstream regulator tool predicts activation of ERK, MEK and CREB1, which is downstream of ERK and MEK. IGF1 can also work through PRKCE to activate the PI3K-AKT pathway [51]. All these signaling components are expressed by our tissues, as compiled in Supplementary Table S5, but only expression of *IGF1* is upregulated, and only in pinguecula.

The other pathway required for epithelial cell proliferation is triggered by extracellular ligands of the FGF family or growth factors that bind ERBB family cell surface receptors: EGF, HBEGF, TGFA and HGF [52]. Supplementary Table S5 also compares expression of genes listed in the ERBB SuperPath of GeneCards Suite. Some of these genes were downregulated in pterygium, but genes encoding the extracellular ligands were upregulated. *HBEGF*, *AREG*, and *AREGB* were upregulated greater than 3-fold in pterygium-NE, while HBEGF was upregulated greater than 3-fold in pterygium-E.

A previous study localized immunoreactive HBEGF protein to the healthy corneal limbal and central epithelium and demonstrated an increased level of protein in the epithelium of pterygium [53]. *AREG* encodes amphiregulin; over-expression in mice causes a psoriasis-like skin phenotype [54]. Significantly, *PSORS1C1*, a gene linked to psoriasis susceptibility, was also upregulated.

Ligand encoding gene *HGF* was highly upregulated in pinguecula. The *HGF* gene product is mainly produced by mesenchymal cells [55] (and not by lymphocytes [56]). It acts on epithelial cells that express the HGF receptor MET [56]. The *MET* gene was expressed in our tissues.

This analysis suggests AREG, AREGB and HBEGF produced by epithelial cells are the main upstream regulators driving epithelial cell proliferation in pterygium. IGF1 produced by epithelial cells and HGF produced by stromal cells appear to be the main upstream regulators driving epithelial cell proliferation in pinguecula. TP53 appears to be functioning to counteract signals for cell proliferation, thus promoting differentiation.

#### 2.8.2. Epithelial Cell Fate

In the comparison between pinguecula and pterygium subgroups, IPA predicted activation of pathways controlled by MYC, TP63 and KLF4 upstream regulators, which control epithelial cell fate. Supplementary Table S6 compares expression of genes in these pathways.

*TP63* encodes a transcription factor that acts together with *PAX6* to specify corneal limbal stem cells [57]. Neither gene changed expression in pterygium or pinguecula. These findings are consistent with a previous study on *TP63* in pterygium [58].

*KLF4*, *MYC* and *POU5F1* encode three of the four transcription factors (along with SOX2) required for generation of induced pluripotent stem cells from mouse embryonic or adult fibroblasts. As each of these factors are present in the corneal limbus, it is likely that they are involved in the maintenance of limbal stem cells, the source of epithelial cells in the mature cornea [59]. We did not detect *SOX2* expression in this study. *KLF4* was modestly upregulated in pterygium-E. *MYC* was moderately upregulated in both pterygium-E and pterygium-NE. *POU5F1* is one of the top upregulated genes in pterygium as is a second transcription factor *PITX1*. Both are expressed in proliferating cells of the corneal epithelial basal layer [59,60].

*KLF5* and *KLF7* [61] encode transcription factors that oppose KLF4 activity; their expression did not change in pterygium or pinguecula. Likewise, *EHF*, encoding a transcription factor thought to interact with *KLF4*/*KLF5* to promote corneal epithelial differentiation [62].

*KLF10* encodes a transcription factor that acts through TGFB signaling to inhibit epithelial cell proliferation and apoptosis [63]. Its expression was upregulated in pterygium. We assembled all genes expressed in our tissues encoding TGF-betas and related gene families encoding BMPs. Elevated expression of *BMP6* was previously shown in pterygium [64] We observed modest upregulation of *BMP2* in both pterygium subtypes, but *BMP6* was upregulated only in pterygium-NE.

Transcriptional inhibitor *SNAI1* is involved in induction of epithelial to mesenchymal transition (EMT) and fibrosis [65]. *SNAI1* was upregulated pinguecula. This suggests that its prediction by IPA is inhibited when comparing pinguecula and pterygium-E and this may be due to the absence of fibrovascular proliferation in our pterygium specimens. *SNAI1* was one of the top upregulated DEGs in pterygium. A previous study detected immunoreactive SNAI1 protein throughout the epithelium of pterygium, but not healthy corneal epithelium [66].

This analysis suggests an expansion of cells from the corneal limbal epithelial compartment in pterygium, and identifies *KLF4*, *MYC* and *POU5F1* and *PITX1* as specifically involved. An increase in EMT in corneal epithelium in pterygium is also suggested, regulated by KLF7, *BMP2*, *BMP6*, and *SNAI1*.

Multiple pathways for epithelial differentiation converge on TP63, including Notch and Wnt [67]. In Supplementary Table S7, we compile some of the genes from the Notch Signaling SuperPath and the Signaling by Wnt SuperPath listed in GeneCards Suite. Some of the Notch pathway genes exhibited a modest change in expression; however, *HES5*, which encodes a transcription factor, was highly upregulated in pterygium.

*WNT7A*, which encodes an extracellular ligand of the Wnt pathway, was reported to control corneal epithelial differentiation through *PAX6* [57]. *WNT7A* expression did not change in our datasets; however, two other genes encoding Wnt ligands—*WNT7B* and *WNT9A*—were upregulated in pterygium. *PPM1N*, which encodes a phosphatase, was one of the top upregulated DEGs in pterygium. The Gene Ontogeny Resource (GO) indicates that one of its functions is positive regulation of the canonical Wnt signaling pathway.

*SFRP2* and *LGR5* were among the top 25 upregulated genes in pinguecula. They function as negative regulators of Wnt signaling in B-cells [68,69]. Moreover, upregulated specifically in pinguecula, *SFRP4*, is a negative regulator of Wnt signaling in T-cells [70]. Upregulation of these genes is likely due to lymphocyte infiltration of our pinguecula specimens.

This analysis suggests increased activity of the Notch and Wnt epithelial differentiation pathways in pterygium, and identifies *HES5*, *WNT7B*, *WNT9A*, and *PPM1N* as being specifically involved.

### 2.9. Tissue Differentiation and Molecular Pathology

#### 2.9.1. Epithelial Differentiation

Keratin genes, the individual units of intermediate filaments expressed by epithelial cells, are a family of 54 different genes, about half being restricted to the hair follicle [71,72,73]. They form obligate heterodimer pairs consisting of type I and type II molecules at equimolar amounts [74]. Keratins define cells as “epithelial” and their expression patterns characterize cells of different tissues and distinguish stages of cell differentiation (4, 8). Expression of specific keratins define the signature for certain cell clusters described above (Table 6 and Supplementary Table S4).

Transcripts for 25 keratin genes and 28 keratin pseudogenes were identified in our datasets. Supplementary Table S8 compiles differential expression patterns for the genes, which are listed in subgroups based on expressing epithelial cell type and differentiation stage, according to Moll [72] and Bragulla [73]. Expressing locations in ocular surface epithelia and skin are according to Wistow [22] and Kao [75]. In general, we observed upregulation of corneal, limbal and conjunctival keratins in pterygium, but down-regulation in pinguecula. This suggests more squamous cell layers in our pterygium specimens, as we noted in the discussion of the cell type signatures above.

Several keratin genes were variably expressed in our specimens. This includes the simple epithelial keratin genes *KRT23* and *KRT24*. In one previous microarray study, *KRT24* was identified as upregulated in pterygium [19]; however, we could not confirm this in independent specimens, suggesting a variable expression. In a second microarray study, *KRT24* was highly upregulated [20]. Moreover, variably identified were genes for several keratins not previously reported to be expressed in the ocular surface epithelia, to our knowledge. This includes *KRT78* and *KRT80*, which encode keratins characteristic of tongue epithelium. The mixed expression of two genes from the subgroup of hard epithelial keratins characteristic of hair and nails, *KRT31* and *KRT40*, was also observed. Their regulation could relate to the patchy skin-type differentiation seen on rare occasions in pterygium [26]. Expression of the unclassified *KRT222* was also identified.

*KRT9* was one of the top upregulated DEGs in this study. Unique among all the other keratin genes expressed by our tissues, it was upregulated at high levels across pterygium and pinguecula. *KRT9* encodes a member of the hyperproliferative subgroup of keratins, expressed by differentiating suprabasal cells [76]. Its expression is highly specific for the specialized palmoplantar epidermis of the palms of the hands and the soles of the feet [72]. Expression of *KRT9* has been reported in hyperproliferative skin diseases outside of the palms and soles [76], but we have not found any previous reports of expression at the ocular surface.

Mutations in *KRT9* cause a rare inherited disease called palmoplantar keratoderma, which manifests as hyperproliferation. Because of this, we looked for altered expression of other genes known to cause the same disorder. Significantly, *TRPV3*, which causes a form of the disease known as Olmstead Syndrome [77], was also strikingly upregulated in both pterygium and pinguecula.

*TRPV3* encodes a member of the transient receptor potential (TRP) cation channel family. Many other family members are also expressed at the ocular surface epithelia [78,79]. Supplementary Table S10 compiles differential expression data all TRP gene family members expressed by tissues analyzed in this study. We observed downregulation of most of these TRP genes in pterygia. In contrast, *TRPV3* was highly upregulated in both pterygium subtypes, and in pinguecula. We note that *TRPV2* was also highly upregulated in pinguecula, but this is likely because it is expressed by lymphocytes [80].

These novel findings indicate aspects of a palmoplantar phenotype which are assumed in pterygium and pinguecula.

#### 2.9.2. Keratinization and Cornification

Several genes involved in formation of the cornified envelop—*CSTA*, *IVL*, *SCEL*, *SPRR3*—are represented in the “Inflammation and Immune Response” category discussed above. *IVL*, *SPRR3* and a third cornification gene, *CAPN14*, are among the top 25 upregulated DEGs identified in this study. *CAPN14* is one of a unique set of genes expressed during cornification in the mucosal epithelium of esophagus, distinguishing it from the dry epidermis of skin [81].

The processes of keratinization and cornification in skin leads to the formation of the outermost skin barrier, a process regulated by pathways that also promote inflammation [82]. Inflammatory disease in skin increases the degree of cornification [83,84]. Inflammatory disease at the ocular surface leads to cornification as well, and is linked to squamous metaplasia, a hallmark of pterygium (Supplementary Table S1) [85]. Squamous metaplasia has been defined as the pathological transition of a nonkeratinized, stratified epithelium into a nonsecretory, keratinized epithelium [86]. This is an accurate description for the changes that occur in severe ocular surface diseases such as Stevens-Johnson Syndrome and vitamin A deficiency, in which transformation to a skin phenotype with keratinization is observed [87]. Squamous metaplasia in dry eye is primarily characterized by cornification [88].

Supplementary Table S10 lists a selection of genes comprising the Keratinization SuperPath in GeneCards Suite, as well as keratins characteristic of conjunctiva. We found that many of the genes expressed in our tissues were among them. The most-upregulated were those that contribute to the cornified envelop and desquamation. These were all upregulated in pterygium, with the highest expression in pterygium-NE. Genes representing desmosome assembly, cornification, and desquamation were consistently downregulated in pinguecula. The highly elevated signal for desquamation genes *PI3* and *KLK7* in pinguecula is likely due to lymphocyte infiltration of the tissues, since expression of these genes is characteristic of this cell type.

An early microarray study identified *SPRR3*, *SPRR1A*, *SPRR1B* and *IVL* from the cornification subcategory as upregulated genes in pterygium [18]. A second study identified *PERP* and *DSP* as genes from the desmosome assembly subcategory [22]. Significantly, no change in expression of genes encoding proteins involved in keratin aggregation was observed in either of these studies. In our study, we found only minimal changes, with modest upregulation of KRT10 in pterygium and no expression of KRT1 in either pterygium or pinguecula; in addition, FLG2 was modestly upregulated in pinguecula. The conjunctival keratins K4 and K14 that are downregulated in keratinization were upregulated in pterygium and were unchanged in pinguecula.

Expression of transcription factor *PAX6* was significantly downregulated in corneal epithelial cells isolated from the severe ocular surface inflammatory diseases of Stevens-Johnson Syndrome and recurrent pterygium [89], and from Sjögren’s syndrome [90]. *PAX6* was downregulated due to inflammation in a Sjögren’s syndrome model, the Aire mouse, and this led to keratinization [91]. In our study, we found no change in expression of *PAX6* in pterygium or pinguecula, consistent with the relative lack of keratinization.

#### 2.9.3. Mucosal Differentiation

Mucins are a family of high molecular weight, heavily-O-glycosylated proteins characteristic of wet (mucosal) epithelia. Membrane-associated mucins (MAMs) that accumulate at the apical cell layer of the ocular surface epithelia are the defining molecules of the mucosal glycocalyx [92,93]. Secreted mucins are produced by conjunctiva, conjunctival goblet cells or lacrimal glands. They can assemble into large molecular weight complexes via disulfide bonds, mixing with the tears to form a mucoaqueous gel.

The 18 mucin genes expressed by the healthy conjunctival epithelium are listed in Supplementary Table S11, as referenced [92,93]. Also included is MUC6, the expression of which occurs in the lacrimal gland. Transcripts from 15 of the genes expressed by healthy conjunctival epithelium were represented in our datasets. Expression of 9 of these genes changed in pinguecula and/or pterygium.

*MUC1*, *MUC4*, *MUC16*, *MUC20* and *MUC21* are the major genes expressed by the ocular surface epithelia encoding MAMs. We found upregulation of these genes in pterygium. Expression of *MUC20* and *MUC21* was decreased in pinguecula. Expression of *MUC3A* has previously been described as very low level in conjunctival epithelium; we observed a further decrease in both pterygia and pinguecula. This analysis indicates that mucosal differentiation is maintained in pterygium, despite epithelial cornification.

The largest changes were in the secreted mucin subgroup. *MUC2* expression was down-regulated in both pterygium subtypes. *MUC7* expression was downregulated in pinguecula, and even more in pterygium-NE, and was one of the top downregulated DEGs in pterygium-E. *MUC6* was the most upregulated of the secreted mucin genes, and increased in both pinguecula and pterygia. This suggests an increased representation in the lesions by a usually minor ocular surface glandular epithelial cell type.

*MUC5AC* is a marker for goblet cells, which secrete the encoded mucin as a major product. We observed increased *MUC5AC* expression in pinguecula, but not in pterygium. An early microarray study reported increased expression of *MUC5AC* and the related *MUC5B* in pterygium [18], while another study reported almost total loss of *MUC5AC* in pterygium, as assessed by immunostaining [85]. This finding is consistent with the variability of epithelial goblet cells in pterygium (Supplementary Table S1).

#### 2.9.4. Fibrovascular Proliferation

One of the variable features of pterygium and pinguecula is fibrovascular proliferation beneath the epithelial component (Supplementary Table S1). Differential expression of genes encoding hemoglobin subunits was identified in several other microarray studies, sometimes downregulated (e.g., [18]) and sometimes upregulated (e.g., [20]). In this study, *HBA1*, *HBA2*, and *HBB* were among the top 25 upregulated DEGs in pinguecula and top 25 downregulated DEGs in pterygium. They are compiled with other genes involved in the process of angiogenesis in Supplementary Table S12. Also highly upregulated in pinguecula (one of the top 25 upregulated DEGs) was *PI16*, a protease inhibitor expressed by vascular endothelial cells and fibroblasts that regulates vascular permeability [94,95]. These genes were downregulated in pterygium, which suggests that our specimens have an atrophic non-vascularized phenotype (Supplementary Table S1). Intriguingly, expression of several regulators of angiogenesis was elevated, including *VEGFA*.

A selection of genes expressed in our tissues encoding extracellular matrix and integrin receptor subunits is compiled in Supplementary Table S13. Examples of these genes are reported as DEGs in several of the gene profiling studies published for pterygium. In one of the earliest studies [18], expression of *COL1A1*, *COL1A2*, *COL3A1*, *COL4A1*, *COL6A3*, *COL15A1*, *FN1*, *POSTN* and *SPARC* was increased in primary pterygium; however, these genes were uniformly downregulated in recurrent pterygium [18]. These same genes were also downregulated in our pterygium samples, consistent with an atrophic phenotype.

Matrix Metalloproteinases (MMPs) are zinc-dependent proteinases involved in all aspects of development and normal bodily processes, and they play a role in almost every disease [96,97]. The 24 human genes of the MMP family are listed in Supplementary Table S14. MMPs are inhibited by Tissue Inhibitors of Metalloproteinases (TIMPs) [97]; the 4 genes of this family are also listed. For comparison, we also list MMPs that we previously determined were upregulated during corneal epithelial repair in mouse [98].

Thirteen MMPs and 3 TIMPs were expressed in our pterygium and pinguecula specimens. *MMP3* was the only MMP/TIMP gene upregulated in pterygium, and only modestly, while *MMP2*, *MMP7*, *MMP10*, and *MMP25* were downregulated. More MMP/TIMP upregulation was observed in pinguecula: *MMP2*, *MMP10*, and *MMP16*, as well as *TIMP2* and *TIMP3*. These findings are consistent with the relative degree of fibrovascular marker gene expression in our pterygium and pinguecula tissue specimens.

## 3. Discussion

Gene expression profiling is an unbiased approach which can provide clues for hypothesis development. Here we report one of the first studies to use RNA-seq technology to comprehensively profile gene expression in pterygium. In addition, we believe this is the very first report of gene expression profiling, of any kind, for pinguecula. We compared pterygium and pinguecula specimens to normal healthy conjunctiva, as well as to one another. We then analyzed the differential gene expression data using various computational analysis tools and by accessing information from various databases and the scientific literature. We confirmed many previous observations about gene expression in pterygium, while also making several intriguing new observations. Our findings have led to specific hypotheses, setting the stage for follow-up in laboratory bench investigations.

### 3.1. Evidence of Genomic Instability and Downregulation of Tumor Suppressor Genes

A challenge for interpretation of gene expression profiling data comparing pterygium and pinguecula specimens is that these lesions have much in common pathologically, but presentation of these common features can be highly variable [26,27,28]. For this reason, it is difficult to know what is a true difference between the two pathologies, and what is only a subtype difference. For example, pterygium and pinguecula variably present with leukocyte infiltration and fibrovascular proliferation (Supplementary Table S1). Consistent with this, we found markers of lymphocyte infiltration and fibrovascular proliferation in our pinguecula specimens but not in our pterygium specimens. Our comparison of DEGs for pinguecula or pterygium revealed a difference of 5% between the two lesions. However, this difference is likely inflated with respect to the true difference in pathology, due to the fact that we compared morphologically different subtypes.

As a way to evaluate gene expression variability among specimens of the same lesion type, we split our pterygium specimens into two groups based on differential amounts of solar exposure. Expression of two major categories of genes varied between the two groups. The first group comprised genes involved in mitotic checkpoints and DNA repair. Differential expression of some DNA repair genes in pterygium vs. conjunctiva has been reported in previous gene expression profiling studies [44,45]. The second group comprised genes involved in inflammation and the immune response. Comparison of pterygium samples based on degree of solar exposure has never been made to our knowledge, and more studies will be needed before any conclusions can be drawn about the validity of association with solar exposure of gene expression differences. However, identification of these two groups of genes as variably expressed among pterygium specimens was itself an important finding. Variable expression of these genes was also observed in pinguecula, but to a lesser extent.

The National Cancer Institute’s Dictionary of Cancer Terms defines genomic instability as “the increased tendency for DNA mutations (changes) and other genetic changes to occur during cell division” (https://www.cancer.gov/publications/dictionaries/cancer-terms/def/genomic-instability; access date: 4 November 2021). Genomic instability is caused by defects in certain processes that control the way cells divide, and involves genes encoding DNA damage repair proteins and genes encoding proteins involved in DNA and chromosome replication. This aptly describes our first group of DEGs, and their differential expression is consistent with the variable pathological characteristics of epithelia atypia and dysplasia ([43] and Supplementary Table S1).

Genomic instability is a hallmark of cancer but recent findings have revealed that it begins in precancerous stages [99]. Pterygium has been suggested to be a premalignant condition that can progress to neoplasia in some cases [100,101,102]. This is supported by the IPA analysis we performed here, which overwhelmingly linked both pterygium and pinguecula with cancer. This may be a key difference between pterygium and pinguecula, as the latter does not exhibit premalignant pathological characteristics.

The genome of eukaryotic cells is particularly at risk during the S phase of the cell cycle when DNA replication occurs [103]. DNA replication can be challenged by exogenous or endogenous events that impede the rate and fidelity of DNA synthesis, collectively referred to as replication stress. Exogenous events include DNA damage due to ultraviolet (UV) light exposure, thought to be a major factor in the pathogenesis of pterygium and pinguecula. DNA damage activates mitotic checkpoints and the DNA repair response, which may or may not be fully successful. Inflammation can arise from the pathological accumulation of genomic DNA fragments in the cytoplasm, a byproduct of the DNA repair response, which then further exacerbates genomic instability [103]. Identification of our second group of DEGs suggest this mechanism may also be operative in both pterygium and pinguecula.

Over the past 30 years, it has become increasingly clear that there is an order to cancer-driver gene mutation [99]. For example, the first event in most colon cancers appears to be mutations that inactivate the *APC* gene, a negative regulator of Wnt signaling, leading to development of benign adenomas with mild dysplasia. This is followed by mutations in other growth controlling genes, increasing dysplasia to moderate or severe levels. Subsequently, inactivation of the *TP53* tumor suppressor gene and mutations in other oncogenes are associated with progression from adenoma to carcinoma. Pterygium usually evolves very slowly and the lesion remains superficial and non-invasive. However, failure to repair DNA could lead to mutations on rare occasions that activate cancer-driver genes, exacerbating replication stress [99].

Analysis of the genomes of thousands of human cancers has revealed that *TP53* is inactivated by mutation in over 50% of sporadic human tumors [104]. At one time, the main pathway for development of pterygium was believed to be mutations in *TP53* [105]. However, this hypothesis has fallen out of favor for a variety of reasons, an important one being that inactivating *TP53* mutations could not be detected [106,107,108]. Although we did not analyze TP53 protein levels in the current study, we found evidence of TP53 activity, as an expression of downstream target *CDKA1A*, which promotes mitotic arrest, increased in pterygium. In addition, expression of mitotic marker *MKI53* was inhibited, consistent with suppression of cell proliferation. Thus, if mutations in cancer-driver genes are involved in the development of pterygium and pinguecula, genes activated earlier in the progression from precancerous to cancerous lesions are more likely [107]. Our findings provide the rationale for a DNA sequence analysis study to identify specific causative mutations, as has been done for human cancers.

Among our top downregulated genes, we identified several genes encoding tumor suppressors, reduced expression of which might help to drive development of pterygium and pinguecula. Arguably the most significant of these is *C10orf90*. The gene is located on the chromosome at a specific region of genomic instability known as a common fragile site, susceptible to deletion in cells under replicative stress. It is a promising candidate for disease-promoting mutation due to solar irradiation. *RARRES1*, *SCGB3A1*, *DMBT1* were also downregulated. *RARRES1* and *SCGB3A1* were identified in previous pterygium gene profiling studies, while *C10orf90* and *DMBT1* are new. All 4 genes were downregulated in pterygium and *SCGB3A1* and *DMBT1* downregulation was validated in independent specimens. *C10orf90* and *RARRES1* were also downregulated in pinguecula.

### 3.2. Limbal Stem Cell Origin Theory and Pathways Controlling Epithelial Cell Proliferation

In further analyzing our data, it was necessary to take into consideration that pterygium and pinguecula lesions are comprised of several different tissues, each composed of different cell types, including epithelial cells, fibroblasts, vascular endothelial cells, and immune and inflammatory cells. The global expression profile does not give information about which cell type(s) is expressing the differentially expressed gene. This challenge was addressed by utilizing cell-type signatures recently identified by scRNA-seq [48,49]. Based on this analysis, we concluded that most of the previously identified epithelial and stromal cell types were present in our specimens. Moreover, information gleaned from the literature enabled us to assign various other genes expressed to specific cell types.

Cell type signatures identified included TP63-expressing limbal stem cell types previously reported to reside in clusters at the head of the pterygium [26]. First identified by Fuchs more than a century ago, the clusters have been named “Fuch’s flecks” [109]. Their presence has been cited in support of the notion that pterygium is a disease of limbal stem cells [26]. Comparing pinguecula and pterygium, IPA predicted activation of pathways controlled by upstream regulators TP63, MYC and KLF4, transcription factors that control epithelial cell fate. We found that genes encoding transcription factors *MYC*, *KLF4*, *POU5F1* and *PITX1* were upregulated in pterygium. These genes are known to be expressed in proliferating cells of the corneal epithelial basal layer [59,60], suggesting an expansion of cells from the corneal limbal epithelial compartment. *MYC*, *KLF4* and *POU5F1* encode three of the four transcription factors (along with SOX2) required for the generation of induced pluripotent stem cells from mouse embryonic or adult fibroblasts [110]. Significantly, however, signatures for quiescent stem cell types of the epithelium that serve as a reservoir for cell expansion were not detectable in our datasets. This is consistent with the limbal stem cell origin theory of pterygium pathology.

IPA upstream regulator analysis indicated that the major pathways for epithelial cell proliferation were differentially activated in pterygium and pinguecula specimens analyzed in this study. Based on their upregulation, AREG, AREGB and HBEGF produced by epithelial cells appeared to be driving epithelial cell proliferation in pterygium. IGF1 produced by epithelial cells and HGF produced by stromal cells appeared to be the main upstream regulators driving epithelial cell proliferation in pinguecula. Further studies using a large number of samples will be necessary to confirm that these pathways distinguish pterygium and pinguecula; however, this is a promising lead.

### 3.3. Stratification, Hyperproliferation, Cornification and Mucosal Differentiation

Data analyzed in this study presented a picture of a stratified and well-differentiated epithelium in both pterygium and pinguecula. Gene expression signatures for all of the basal and differentiated cell types that compose the ocular surface epithelial were represented. Our further analysis of pathways controlled by upstream regulator TP63 suggested increased activity of the Notch and Wnt epithelial differentiation pathways in pterygium, and identified *WNT7B*, *WNT9A*, *PPM1N* and *HES5* as likely involved.

In general, we observed upregulation of corneal, limbal and conjunctival cell-type markers in pterygium, suggesting an expansion of these cell types. Upregulation of keratin genes associated with hyperproliferation was also seen. Our further analysis of pathways controlled by upstream regulator TP63 suggested increased EMT in pterygium, regulated by *BMP2*, *BMP6*, *SNAI1* and *KLF7*. *SNAI1* was one of the top upregulated genes in pterygia. EMT plays a critical role in tumor progression and malignant transformation, endowing the incipient cancer cell with invasive and metastatic properties [111]. However, partial EMT occurs as a normal physiological response to injury in squamous epithelia. Cells acquire an intermediate phenotype known as “metastable”, which allows them to move while maintaining loose contacts rather than migrating as individual cells [111]. An increase in EMT has also been linked to hyperproliferation of squamous epithelia [112]. These findings are consistent with a migratory front and squamous metaplasia, two common features of pterygium.

Epithelial cell-type signature genes were relatively more downregulated in our pinguecula specimens, suggesting a thinning of the epithelium in these specimens. Downregulation of keratin genes associated with hyperproliferation was also seen. The exception was, *KRT9*, which encodes a hyperproliferative-type keratin upregulated in both pterygium and pinguecula—in fact, it was among the top upregulated genes of this study. Its expression at the ocular surface has not been reported previously. *KRT9* is a marker for suprabasal cells and is highly specific for the specialized palmoplantar epidermis found on the palms of the hands and the soles of the feet [72]. Mutations cause palmoplantar keratoderma, a rare inherited disease that manifests as hyperproliferation. Significantly, *TRPV3*, which causes a form of the disease known as Olmstead Syndrome [77], was also highly upregulated in both pterygium, and moderately so in pinguecula.

*TRPV3* is a member of the transient receptor potential (TRP) cation channel family. Unlike *KRT9*, *TRPV3* expression is not specific for palmoplantar skin, and expression has been described in the ocular surface epithelia [78,79]. TRP channels function to control a variety of processes, including epithelial cell proliferation, temperature, itch and pain sensation, and vasoregulation. In skin keratinocytes, TRPV3 forms ion channels with TRPV1 and associates with EGFR and one of its ligands, TGFA, creating a functional signalosome. Over-expression in skin results in the development of a hyperkeratotic inflammation, with a cellular profile much like atopic dermatitis [113]. This suggests that TRPV3 expression could contribute to the proliferative and inflammatory phenotype we observed in our pterygium and pinguecula specimens.

Squamous metaplasia has been defined as the pathological transition of a nonkeratinized, stratified epithelium into a nonsecretory, keratinized epithelium [86]. This is an accurate description of the changes that occur in severe ocular surface disease such as Stevens-Johnson Syndrome and vitamin A deficiency, where transformation to a skin phenotype with keratinization is observed [87]. However, squamous metaplasia in inflammatory diseases like dry eye is primarily characterized by cornification [88]. Several genes involved in formation of the cornified envelop: *IVL*, *SPRR3* and *CAPN14*, were among the top upregulated genes identified in this study. Other genes distinguishing cornification were also upregulated. In contrast, we found minimal changes in expression of genes associated with keratinization in pterygium or pinguecula. This included no change in expression of transcription factor *PAX6*, downregulation of which has been linked to keratinization at the ocular surface [89,90,91].

MAMs, which accumulate in a polarized manner on apical cell layer of the ocular surface epithelia, are the defining molecules of the mucosal glycocalyx [92,93]. We found modest upregulation or downregulation of MAM genes in pterygium and pinguecula, but overall MAM expression was maintained, consistent with continued mucosal differentiation.

Larger changes were observed in the expression of genes encoding specific secreted mucin produced by conjunctiva, conjunctival goblet cells or lacrimal gland. *MUC7* expression was downregulated in pinguecula and was one of the top downregulated genes in pterygium. *MUC2* expression was down-regulated in pterygium and pinguecula. In contrast, *MUC6* was highly upregulated in pterygium, and moderately so in pinguecula. This suggests increased representation in the lesions by a glandular epithelial cell type, and is consistent with the increased expression of simple epithelial keratin genes, which were also observed.

MUC5AC is a marker for goblet cells, which secrete this mucin as a major product [92,93]. MUC5AC protein can assemble into large molecular weight complexes via disulfide bonds, mixing with the tears to form a mucoaqueous gel. We observed increased MUC5AC expression in pinguecula, but not in the pterygia samples. This finding is consistent with the variable presence of epithelial goblet cells observed in pterygium.

### 3.4. Fibrovascular Proliferation and MMPs

As noted above, one of the variable features of pterygium and pinguecula is fibrovascular proliferation beneath the epithelial component, and in our study, we found evidence of fibrovascular proliferation in our pinguecula specimens but not in our pterygium specimens. Expression of signature genes for cells distinguishing blood vessels, limbal vessels and melanocytes were upregulated in pinguecula, but downregulated in pterygium. Upregulation of markers for fibrovascular proliferation were also seen in pinguecula only. For example, hemoglobin genes *HBA1*, *HBA2*, and *HBB* were among the top 25 upregulated genes in pinguecula and top 25 downregulated genes in pterygium. All genes encoding extracellular matrix components were downregulated in pterygium, but many of them were upregulated in pinguecula. Perplexingly, while molecular markers indicate that our pterygium specimens were not vascularized, expression of several regulators of angiogenesis was elevated, including *VEGFA*.

Demonstration of the role of MMPs in tissue invasion in cancer in the 1990s suggested a possible similar role in pterygium and many studies were published on MMP expression in pterygium during this period (reviewed in [107]). Unlike cancers, however, neither pterygium nor pinguecula are invasive lesions. In fact, microarray gene profiling studies of pterygium have not typically identified differentially expressed MMP genes (e.g., [18]). A recent microarray study [20] found *MMP9* to be highly upregulated, but this gene is expressed by leukocytes; thus, upregulation could be due to leukocyte infiltration. Thus, our findings suggest that MMP/TIMP expression is one of the variable characteristics of pterygium.

## 4. Materials and Methods

### 4.1. Research Subjects

All the procedures of this study were carried out as stipulated in the Good Practice Guidelines for Clinical Research in Human Beings of the Ministry of Health (guide 1480/2011), the Declaration of Helsinki, and Provincial Law 9694. The confidentiality of the data is protected in accordance with Law 25326/2000 on the Protection of Personal Data–Habeas Data. The study was reviewed and approved by the Institutional Committee for the Ethical Evaluation of Health Research of the Hospital Nacional de Clínicas.

Individuals of both sexes who were preparing to undergo simple excision surgery to remove pterygia or pingueculae were enrolled in the study. Patients preparing for cataract or retinal detachment surgery were also enrolled to obtain healthy conjunctival specimens as controls. All patients were informed about the surgical technique and procedures required, as well as the potential benefits and complications. All patients signed detailed informed consent prior to surgery.

The inclusion criterion for pterygia was a lesion of grade 2 or 3 according to the classification system described by Sheppard and colleagues [114], with or without symptomatology, unipolar or bipolar. Exclusion criteria were: (1) eye surface diseases such as limbal conjunctival alterations; (2) conjunctival degeneration of all types; (3) dry eye; (4) ocular pemphigoid; (5) conjunctival tumor lesions of all types; (6) previous surgical history with conjunctival manipulation; (7) conjunctival and conjunctival-corneal trauma; and (8) systemic treatment with nonsteroidal corticosteroids or anti-inflammatory drugs at the time of surgery.

Work activities of pterygium patients enrolled in this study implied differential exposure to UVR. These activities were: farmers and livestock keepers (highly exposed to UVR) or merchant, housewife, and public employee (normally exposed to UVR).

### 4.2. Clinical Specimens

All surgeries were performed in the same operating room at IMOC, Córdoba, Argentina. Eyes were anesthetized with topical Proparacaine HCL 0.5% (Poen-caina^®^, POEN laboratory, Argentina) followed by subconjunctival injection of lidocaine/epinephrine.

For pterygium, the head and part of the body of the lesion was dissected with Westcott scissors and a simple conjunctival closure was performed. Pinguecula presenting as a whitish-yellowish degenerative growth of the conjunctiva, near the corneal limbus in the interpalpebral fissure on the nasal or temporal sector, was diagnosed by biomicroscopy and surgically resected. Samples of healthy conjunctiva were taken from individuals at the time of cataract surgery or retinal detachment surgery. Bulbar conjunctiva biopsies were obtained at 2 mm above the corneal limbus, in the upper temporal quadrant.

All tissues were carefully handled with a tooth-free clamp. They were placed in an Eppendorf tube containing Invitrogen RNA*later* Stabilization Solution (Thermo Fisher Scientific, Waltham, MA, USA) and stored at −80 °C for further analysis.

### 4.3. RNA-Seq

Altogether, 10 pterygia, 7 pingueculae, and 7 normal conjunctival samples, were collected for RNA-seq. RNA was purified according to the manufacturers protocol provided with the Invitrogen RNA*later* Stabilization Solution. RNA-Seq was performed in the Next Generation Sequencing Core at the University of Minnesota Genomics Center (Twin Cities, MN, USA).

Total RNA was quantified using a fluorimetric RiboGreen assay. RNA integrity was assessed using capillary electrophoresis (e.g., Agilent BioAnalyzer 2100), generating an RNA Integrity Number (RIN). The criteria for samples to pass the initial QC step were a yield of ≥500 ng of RNA and an RIN of 8 or greater. Samples that advanced to the next step were split into 4 groups: conjunctiva (2), pinguecula (2), pterygia-NE (2), which included specimens from patients with outdoor work activities and pterygia-NE (2), which included specimens from patients with indoor work activities.

RNA samples were converted to sequencing libraries using the Illumina Truseq Stranded mRNA Sample Preparation Kit (catalogue #RS-122-2103, Illumina, Inc., San Diego, CA, USA), according to the manufacturer’s protocol. Briefly, 500 ng of total mRNA was purified using oligo-dT coated magnetic beads, fragmented and then reverse transcribed into cDNA. The cDNA was adenylated and then ligated to dual-indexed (barcoded) adaptors and amplified using 15 cycles of PCR. The final library size distribution was validated using capillary electrophoresis and quantified using fluorimetry (PicoGreen). Indexed libraries were then normalized, pooled and size-selected to 320bp +/− 5% using Caliper’s XT instrument.

Truseq libraries were hybridized to a paired end flow cell and individual fragments were clonally amplified by bridge amplification on the Illumina cBot. Once clustering was complete, the flow cell was loaded on the HiSeq 2500 and sequenced using Illumina’s SBS chemistry. Upon completion of read 1, an 8 bp index read for Index 1 was performed. The Index 1 product was then removed and the template re-anneals to the flow cell surface. The run proceeded with 7 chemistry-only cycles, followed by an 8 bp index read to read Index 2. Finally, the library fragments were resynthesized in the reverse direction and sequenced from the opposite end of the read 1 fragment, thus producing the template for the paired end read 2.

Base call (.bcl) files for each cycle of sequencing were generated by Illumina Real Time Analysis (RTA) software. The base call files and run folders were then exported to servers maintained at the Minnesota Supercomputing Institute. Primary analysis and de-multiplexing were performed using Illumina’s bcl2fastq software 2.20. The result of the bcl2fastq workflow was de-multiplexed FASTQ files that were used for subsequent analysis by the mapping software and aligner.

#### 4.3.1. Differential Gene Expression Analysis

Raw sequence data was processed through PartekFlow RNAseq pipeline (Partek Inc., St. Louis, MO, USA) as follows. A pre-alignment quality assessment/quality control was performed and bases with Q > 30 were retained for analysis. Bowtie2 was used to filter out non-human DNA, mtDNA and rDNA from the samples and STAR 2.5.3 aligner was used to map the high-quality reads to the GRCh37 human genome assembly. Aligned reads were quantified for differential abundance among samples using DESeq2. The *p*-values were adjusted with the Benjamini–Hochberg method for multiple comparison testing. Significance of DEGs was accepted at an adjusted *p*-value lower than 0.05 [24].

#### 4.3.2. Validation by Quantitative PCR (qPCR)

For validation of RNA sequencing results, independent tissue specimens were collected (5 pingueculae, 6 pterygia, and 7 healthy conjunctival controls), for evaluation of a group of 13 randomly selected upregulated or downregulated genes by quantitative PCR (qPCR). At least 4 independent determinations were done for each gene.

RNA was isolated from individual tissue samples using GeneJET RNA Purification Kit (Thermo Fisher Scientific) following the manufacturer’s instructions and using PureLink^®^ DNase Set (Invitrogen, Carlsbad, CA, USA) to remove DNA contamination from columns. First-strand cDNA was synthesized from 1 ug of total RNA using a reverse transcription kit (High Capacity Reverse Transcription Kit; Applied Biosystems, Foster City, CA, USA) following the manufacturer’s instructions.

The qPCR reaction was performed using SYBR^®^ Green reagents (iTaq Universal SYBR Green Supermix, Bio-Rad, Hercules, CA, USA) with the specific primers listed in Supplementary Table S15. The following parameters were used: 30 s at 95 °C, followed by 40 cycles of 5 s at 95 °C and 30 s at 60 °C. All samples were normalized to RNA levels of the housekeeping gene *GAPDH*. The comparative CT method was used for relative quantification, selecting the relative amount in normal conjunctival samples as the calibrator.

#### 4.3.3. Pathway Analysis

Pathway analysis was performed using Ingenuity Pathway Analysis (IPA) software (Qiagen, Mountain View, CA, USA) to identify biological functions and disease categories that are enriched among DEGs. The IPA upstream regulator tool was used to predict activated or inhibited signal transduction pathways.

## 5. Conclusions

A hallmark of cancer, genomic instability begins in the precancerous stage and can be exacerbated by inflammation due to products of DNA damage. Our findings suggest that pterygium and pinguecula are both on the developmental path towards neoplastic transformation, with pterygium being further along this path. However, both lesions are clearly still benign, as evidenced by expression of other genes indicating their well-differentiated and non-invasive character. In the course of this study, we identified a changed expression of other new genes in common to both lesions that provide further insight into pathophysiology. Finally, we identified genes and pathways that may distinguish the two lesions, and suggest novel targets for therapy.

## Figures and Tables

**Table 1:** Transcriptome Differences among Tissue Specimens.

Tissue Comparison	Normal Conjunctiva: 18,630 Genes Identified			% Similarity
	Number Differentially Expressed Genes (DEGs)			
	Upregulated	Downregulated	Total	
Conjunctiva vs. Pinguecula	3015	2074	5089	73%
Conjunctiva vs. Pterygium-E	2564	3238	5802	69%
Conjunctiva vs. Pterygium-NE	2069	4188	6257	66%
Pinguecula vs. Pterygium-E	418	453	871	95%
Pinguecula vs. Pterygium-NE	431	538	969	95%
Pterygium-NE vs. Pterygium-E	61	88	149	99.2%

**Table 2:** Top 25 Genes Upregulated with Respect to Normal Conjunctiva.

Ordered by Pterygium-E: Fold Change				Ordered by Pterygium-NE: Fold Change				Ordered by Pinguecula: Fold Change			
Gene (HUGO Designation)	Pt-E	Pt-NE	Ping	Gene (HUGO Designation)	Pt-E	Pt-NE	Ping	Gene (HUGO Designation)	Pt-E	Pt-NE	Ping
*NPIPA3*	119.47	2.47	3.68	*RP11-386G11.10*	16.95	768.94	−1.21	*IGLC3*	5.67	1.76	239.41
*CLEC18A*	58.77	1.10	4.85	*RP11-78A19.3*	2.91	401.63	2.02	*IGHG1*	−1.17	−1.99	41.06
*PEAK3*	27.64	6.26	3.51	*AC068831.15*	10.72	98.55	−1.35	*IGHA1*	4.00	1.79	38.03
*AC007000.12*	19.84	1.73	1.49	*SPRR3*	2.75	34.38	1.48	*IGLC2*	−1.25	−3.55	34.89
*RP11-386G11.10*	16.95	768.94	−1.21	*IVL*	3.83	28.63	−1.27	*IGHM*	−5.22	−1.93	30.77
*FAM71A*	16.82	−1.92	3.44	*Z95704.2*	1.31	25.33	1.42	*IGKC*	−1.11	−1.32	28.80
*RP1-261G23.7*	13.43	9.05	1.22	*RRAD*	4.81	23.98	−1.16	*PI16*	1.38	−1.09	26.83
*ZBTB45P1*	12.44	−3.93	−3.24	*SLC9A3*	5.85	17.36	1.69	*CD2*	−1.47	−1.72	26.33
*CGB7*	11.39	1.33	3.96	*PPM1N*	5.87	17.06	9.54	*ADH1C*	1.70	1.77	21.56
*TTLL10-AS1*	10.85	9.00	4.52	*AATK*	8.70	15.07	2.94	*IGF1*	−1.18	−2.07	16.78
*AC068831.15*	10.72	98.55	−1.35	*FOSL1*	2.97	14.79	−1.26	*NR2F1*	1.36	1.03	15.39
*RP11-45M22.3*	10.72	4.82	2.67	*FOSL1P1*	1.49	14.27	−1.05	*SFRP2*	2.38	1.43	14.57
*EIF4HP2*	10.53	3.03	1.15	*RP11-556K13.1*	1.05	14.19	−1.29	*IDO1*	−1.58	−1.84	14.39
*PCK1*	10.13	7.64	−1.03	*TMPRSS11B*	1.54	14.01	2.74	*IGHA2*	2.75	−1.09	13.94
*DSPP*	9.99	1.62	3.03	*PALM2-AKAP2*	3.45	13.67	4.19	*PI3*	−1.04	−1.09	13.64
*PITX1*	9.86	8.20	1.36	*ENDOU*	2.07	13.42	−1.62	*HBA1*	−13.53	−14.70	13.58
*RP11-203J24.8*	9.66	9.95	−1.36	*LGI3*	2.30	13.09	1.10	*MYCT1*	−1.21	1.34	13.44
*POU5F1*	9.36	9.09	1.84	*RP5-1142A6.8*	2.13	12.31	3.13	*RP11-513I15.6*	4.70	1.34	13.05
*RP11-164P12.3*	9.34	4.20	2.26	*AC004943.1*	3.71	12.14	4.56	*LGR5*	2.63	−1.55	12.58
*CCDC163P*	9.33	2.10	1.97	*SNAI1*	2.18	11.98	6.09	*STK32A*	2.05	1.37	12.29
*PLD6*	9.29	2.73	1.07	*FABP5P3*	3.40	11.77	1.66	*UCHL1*	1.15	−1.17	12.18
*RP5-1126H10.2*	8.92	1.71	1.27	*LDHAL6B*	4.74	11.71	7.41	*POSTN*	−7.13	−4.82	12.06
*RP11-83B20.1*	8.71	1.62	1.68	*MUC6*	8.45	10.96	2.62	*CCR2*	1.44	−1.67	12.02
*AATK*	8.70	15.07	2.94	*GJC2*	5.93	10.84	3.48	*KRT9*	8.25	4.74	11.86
*C3orf36*	8.55	4.94	2.28	*CAPN14*	4.27	10.73	−2.37	*SLC26A4-AS1*	−1.27	−1.40	11.82
Color Key											
DEGs 5-fold higher than conjunctiva						DEGs 5-fold lower than conjunctiva					
DEGs 4 to-5-fold higher than conjunctiva						DEGs 4 to-5-fold lower than conjunctiva					
DEGs 3 to 4-fold higher than conjunctiva						DEGs 3 to 4-fold lower than conjunctiva					
DEGs 2 to 3-fold higher than conjunctiva						DEGs 2 to 3-fold lower than conjunctiva					
DEGs 1.5 to 2-fold higher than conjunctiva						DEGs 1.5 to 2-fold lower than conjunctiva					

**Table 3:** Top 25 Genes Downregulated with Respect to Normal Conjunctiva.

Ordered by Pterygium-E: Fold Change				Ordered by Pterygium-NE: Fold Change				Ordered by Pinguecula: Fold Change			
Gene (HUGO Designation)	Pt-E	Pt-NE	Ping	Gene (HUGO Designation)	Pt-E	Pt-NE	Ping	Gene (HUGO Designation)	Pt-E	Pt-NE	Ping
*MUC7*	−51.82	−8.48	−2.36	*DMBT1*	−44.67	−125.64	2.78	*CTD-3232M19.2*	−12.34	−43.07	−64.70
*DMBT1*	−44.67	−125.64	2.78	*CTC-432M15.3*	−13.30	−70.91	−39.26	*RP11-552F3.12*	−9.60	−14.10	−48.56
*POLR2J2*	−24.47	−8.51	2.91	*CTD-3232M19.2*	−12.34	−43.07	−64.70	*HSPA6*	2.42	1.23	−45.93
*MRC1*	−16.54	−12.29	−3.42	*RPL36A-HNRNPH2*	−2.96	−26.04	−13.63	*CTC-432M15.3*	−13.30	−70.91	−39.26
*HBA1*	−13.53	−14.70	13.58	*PFN1P3*	−9.92	−25.12	−10.52	*HSPA7*	−1.34	−3.73	−21.74
*EEF1B2P1*	−13.44	−4.77	−4.12	*CCL18*	−11.94	−20.29	−2.50	*AKR1B10*	−2.16	−8.59	−21.73
*CTC-432M15.3*	−13.30	−70.91	−39.26	*WDR72*	−2.75	−18.85	−4.49	*RPL36A-HNRNPH2*	−2.96	−26.04	−13.63
*CTD-3232M19.2*	−12.34	−43.07	−64.70	*SNORA11D*	−3.25	−17.11	−7.69	*CORO7-PAM16*	−1.64	1.93	−12.71
*SCGB3A1*	−12.04	−2.75	2.92	*RPSAP41*	−10.31	−15.95	−8.09	*HSPA1A*	1.24	1.03	−11.17
*CCL18*	−11.94	−20.29	−2.50	*HBA1*	−13.53	−14.70	13.58	*PFN1P3*	−9.92	−25.12	−10.52
*C10orf90*	−11.78	−5.34	−1.66	*RP11-552F3.12*	−9.60	−14.10	−48.56	*RPS3P7*	−2.42	−7.17	−9.62
*RNU1-4*	−11.66	−4.14	−1.61	*MRC1*	−16.54	−12.29	−3.42	*RP11-713P17.3*	−1.74	1.33	−8.70
*RNU1-2*	−11.66	−4.15	−1.61	*FCGR3A*	−4.51	−12.13	−1.06	*AC087392.1*	−2.80	−1.98	−8.14
*RP11-302B13.5*	−11.28	−10.58	−4.57	*FAIM3*	−5.53	−12.05	−1.47	*RPSAP41*	−10.31	−15.95	−8.09
*HBB*	−11.00	−11.87	5.39	*HBB*	−11.00	−11.87	5.39	*RP4-737E23.2*	−2.33	−1.30	−7.96
*HBA2*	−10.64	−10.17	3.76	*USP32P2*	−1.24	−11.60	−2.45	*SNORA11D*	−3.25	−17.11	−7.69
*LINC00623*	−10.49	−4.63	1.74	*RP11-302B13.5*	−11.28	−10.58	−4.57	*RP11-530C5.1*	−4.97	−7.73	−7.27
*RPSAP41*	−10.31	−15.95	−8.09	*ZSCAN23*	−2.78	−10.32	−3.77	*RP3-522J7.5*	−7.58	−4.27	−7.06
*HERC2P5*	−9.98	−9.40	−1.50	*HBA2*	−10.64	−10.17	3.76	*RP11-74C1.4*	1.23	−2.66	−6.98
*PFN1P3*	−9.92	−25.12	−10.52	*TSNAX-DISC1*	−3.35	−10.09	−2.16	*HSPA1B*	1.82	1.34	−6.98
*RP11-552F3.12*	−9.60	−14.10	−48.56	*RARRES1*	−6.19	−9.86	−2.09	*ATP6V1B1*	−5.02	−4.80	−6.95
*S100B*	−9.46	−7.88	2.18	*TAS2R46*	−4.52	−9.55	−1.75	*KRT18P60*	−2.86	−1.66	−6.73
*CYP1B1-AS1*	−9.09	−4.43	−3.49	*AC084219.3*	−6.76	−9.48	−6.29	*ZNF322P1*	−7.09	−2.83	−6.70
*IGHG3*	−9.03	−3.18	10.98	*C3*	−2.83	−9.45	−2.22	*ATP1B1P1*	−4.15	−5.76	−6.68
*RP11-75A9.3*	−8.95	−4.42	−2.45	*HERC2P5*	−9.98	−9.40	−1.50	*RP11-85F14.5*	−3.64	−8.11	−6.40
Color Key											
DEGs 5-fold higher than conjunctiva						DEGs 5-fold lower than conjunctiva					
DEGs 4 to-5-fold higher than conjunctiva						DEGs 4 to-5-fold lower than conjunctiva					
DEGs 3 to 4-fold higher than conjunctiva						DEGs 3 to 4-fold lower than conjunctiva					
DEGs 2 to 3-fold higher than conjunctiva						DEGs 2 to 3-fold lower than conjunctiva					
DEGs 1.5 to 2-fold higher than conjunctiva						DEGs 1.5 to 2-fold lower than conjunctiva					

**Table 4:** IPA Top Diseases and Biological Functions.

Tissue Comparison	Diseases or Functions Annotated	*p*-Value	# Molecules
Conjunctiva vs. Pinguecula	Cancer, Organismal Injuries & Abnormalities	3.63 × 10^−2^ to 7.20 × 10^−7^	105
Conjunctiva vs. Pterygium-E	Cancer, Organismal Injuries & Abnormalities	9.64 × 10^−3^ to 4.80 × 10^−8^	205
Conjunctiva vs. Pterygium-E	Cancer, Organismal Injuries & Abnormalities	7.75 × 10^−3^ to 3.89 × 10^−7^	276
Pinguecula vs. Pterygium-E	Cancer, Organismal Injuries & Abnormalities	2.75 × 10^−6^ to 1.32 × 10^−38^	670
Pinguecula vs. Pterygium-NE	Cancer, Organismal Injuries & Abnormalities	2.57 × 10^−4^ to 1.96 × 10^−31^	721
Pterygium-E vs. Pterygium-NE	Cancer, Organismal Injuries & Abnormalities	4.84 × 10^−2^ to 1.48 × 10^−5^	70
	Dermatologic Diseases & Conditions	4.84 × 10^−2^ to 1.91 × 10^−5^	16
	Immunological Disease	4.89 × 10^−2^ to 3.82 × 10^−4^	12
	Inflammatory Disease	4.84e × 10^−2^ to 3.82 × 10^−4^	13
	Cell Cycle		5

**Table 5:** DEGs in Pterygium-E vs. Pterygium-NE.

Gene (HUGO Designation)	Fold-Change vs. Conjunctiva			Protein Function
	Pterygium-E	Pterygium-NE	Pinguecula	
1. DNA Damage Response, Mitotic Checkpoints, Cell Cycle				
*MKI67*	−1.70	−3.78	1.29	Maintain mitotic chromosomes
*XRCC2*	1.67	−3.28	−2.12	Assists RAD51 to repair DNA damage by homologous recombination
*KIF20A*	1.43	−3.06	1.43	Recruitment of PLK1 to the central mitotic spindle
*GTSE1*	2.30	−2.91	1.57	Mitotic spindle regulator required for G2/M progression
*PLK1*	−1.40	−2.87	−1.68	Involved in mitosis, cytokinesis, DNA repair response
*SKA3*	1.64	−2.27	1.36	Required for mitotic spindle checkpoint silencing for anaphase entry
*XRRA1*	1.04	−2.12	−1.13	X-Ray Radiation Resistance Associated 1
*RAD51D*	1.13	−1.84	−1.47	Double strand break DNA repair protein
*TMPRSS11A*	1.90	−1.77	−1.23	Transmembrane serine protease, induces G1 cell cycle arrest
*RAD51C*	−1.35	−1.70	−1.01	Double strand break DNA repair protein
*SLC26A4-AS1*	−1.27	−1.40	11.82	Inhibits expression of DNA double-strand break repair genes
*RAD51*	−1.45	−1.35	−1.61	Double strand break DNA repair protein
*XAB2*	1.48	−1.26	1.01	Repairs DNA damage by homologous recombination
*RAD50*	−1.09	−1.25	1.16	Double strand break DNA repair protein
*RAD51B*	−1.12	−1.14	1.47	Double strand break DNA repair protein
*SLX1B*	4.72	−1.14	1.14	Endonuclease that functions in DNA repair and recombination
*TP53*	1.11	1.05	−1.04	Mitotic checkpoint regulator, involved in the DNA repair response
*XRCC3*	1.45	1.38	1.63	Assists RAD51 to repair DNA damage by homologous recombination
*FOXF2*	−2.21	2.03	1.47	Transcription factor drives degradation of CTNNB1
*CDKN1A*	1.83	2.17	−1.07	P21 cyclin dependent kinase inhibitor
*PLD6*	9.29	2.73	1.07	Endonuclease thought to be involved in maintaining genomic stability
*C19orf40*	−1.72	2.76	1.18	(FAAP24) DNA damage response resolving crosslinking lesions
*NCCRP1*	−1.19	2.69	−1.08	Tumor suppressor
*TEX14*	1.58	7.54	−1.25	Recruited to kinetochores by PLK1 during early mitosis
*AATK*	8.70	15.07	2.94	Induced during apoptosis, may be needed for growth arrest
2. Inflammation and Immune Response				
*FCGR1A*	1.09	−8.61	−1.27	High affinity Fc-gamma receptor
*SERPINB3*	1.37	−5.78	1.50	Serine proteinase inhibitor
*MKI67*	−1.70	−3.78	1.29	Maintain mitotic chromosomes
*XRCC2*	1.67	−3.28	−2.12	Maintain chromosome stability and repair DNA damage
*TYMP*	−1.27	−2.61	−1.19	Thymidine phosphorylase; promotes angiogenesis
*CPNE1*	1.05	−1.73	−1.63	Copine 1, calcium-dependent membrane binding protein
*LGALS9B*	2.44	−1.44	−1.18	One of the galectins
*MVD*	2.38	−1.19	1.56	Mevalonate diphosphate decarboxylase; cholesterol biosynthesis
*IL1R2*	−5.34	−1.17	−2.57	IL1 Receptor 2
*CLEC18A*	58.77	1.10	4.85	Lectin that functions as a co-receptor for TLR3 (toll-like receptor 3)
*IER2*	4.03	1.50	1.24	DNA binding protein that seems to act as a transcription factor
*OCA2*	−2.92	1.69	−2.97	Melanin synthesis
*CA14*	−2.62	1.85	1.27	Carbonic Anhydrase 14
*TBX1*	−1.51	2.04	1.85	T-box transcription factor
*CSTA*	−1.30	2.05	−1.27	Cystatin A (cysteine proteinase inhibitor)
*SCEL*	1.27	2.14	−1.12	Sciellin (cornified envelope)
*ABCA12*	1.10	2.20	−1.63	Transporter involved in lipid homeostasis
*CCDC144A*	−1.96	2.90	1.83	Coiled coil containing
*FOXF2*	−2.21	2.03	1.47	Transcription factor
*GCNT3*	1.10	2.01	−1.56	Enzyme responsible for O-linked glycosylation in mucins
*IL1RN*	1.91	4.49	−1.76	Interleukin 1 receptor antagonist
*APOBEC3A*	1.96	7.84	−1.38	Apolipoprotein B mRNA editing enzyme
*ENDOU*	2.07	13.42	−1.62	Endonuclease; promotes tolerance via B-cell activation-induced death
*FOSL1*	2.97	14.79	−1.26	Transcription factor involved in stress responses; AP-1 complex
*PPM1N*	5.87	17.06	9.54	Putative Mg2+/Mn2+ Dependent Protein Phosphatase
*IVL*	3.83	28.63	−1.27	Involucrin (cornified envelope)
*SPRR3*	2.75	34.38	1.48	Small Proline Rich Protein 3
Color Key				
DEGs 5-fold higher than conjunctiva				DEGs 5-fold lower than conjunctiva
DEGs 4 to-5-fold higher than conjunctiva				DEGs 4 to-5-fold lower than conjunctiva
DEGs 3 to 4-fold higher than conjunctiva				DEGs 3 to 4-fold lower than conjunctiva
DEGs 2 to 3-fold higher than conjunctiva				DEGs 2 to 3-fold lower than conjunctiva
DEGs 1.5 to 2-fold higher than conjunctiva				DEGs 1.5 to 2-fold lower than conjunctiva

**Table 6:** Changes in Expression of Cell Type Signature Genes in Human.

Gene (HUGO Designation)	Fold Change vs. Conjunctiva			Function
	Pterygium-E	Pterygium-NE	Pinguecula	
Epithelial Cell Types				
Conjunctival Suprabasal (cluster 6)				
KRT4	2.39	4.37	−1.20	Epithelial keratin
KRT13	1.57	3.34	−1.68	Epithelial keratin
MUC1	1.27	1.18	−1.06	Membrane-Associated Mucin
MUC4	2.48	1.09	1.35	Membrane-Associated Mucin
S100A8	−3.04	−1.69	1.04	Calcium binding protein
S100A9	−1.78	−1.38	−1.06	Calcium binding protein
Conjunctival Basal (cluster 0)				
KRT6A	1.64	1.43	−1.48	Epithelial keratin
KRT13	1.57	3.34	−1.68	Epithelial keratin
KRT14	1.17	1.06	−2.04	Epithelial keratin
KRT15	−1.32	1.07	−1.95	Epithelial keratin
S100A8	−3.04	−1.69	1.04	Calcium binding protein
S100A9	−1.78	−1.38	−1.06	Calcium binding protein
Corneal Limbal Superficial (cluster 5)				
KRT24	−3.34	2.63	−1.45	Epithelial keratin
LYPD2	1.20	3.08	1.06	LY6/PLAUR Domain Containing 2
Corneal Limbal Suprabasal and Superficial (cluster 2)				
KRT3	2.88	2.13	−1.53	Epithelial keratin
KRT12	1.28	−1.67	−1.84	Epithelial keratin
KRT24	−3.34	2.63	−1.45	Epithelial keratin
AREG	1.77	5.03	1.40	EGF family amphiregulin
Corneal Limbal Suprabasal (cluster 7)				
KRT3	2.88	2.13	−1.53	Epithelial keratin
KRT12	1.28	−1.67	−1.84	Epithelial keratin
KRT24	−3.34	2.63	−1.45	Epithelial keratin
Corneal Limbal Suprabasal (cluster 4)				
KRT14	1.17	1.06	−2.04	Epithelial keratin
KRT15	−1.32	1.07	−1.95	Epithelial keratin
GJA1	−1.27	−1.57	−1.31	Gap junction connexin
CLDN1	−1.16	−1.03	−1.63	Tight junction claudin
CLDN4	2.49	3.20	−1.67	Tight junction claudin
TP63	1.22	1.40	−1.28	Transcription factor p53 family
Corneal Limbal Basal and Suprabasal (cluster 1)				
KRT12	1.28	−1.67	−1.84	Epithelial keratin
GJB6	1.72	−1.22	−1.03	Connexin (gap junction)
HES1	1.04	−1.32	−1.13	Notch signaling transcription factor
HES5	6.02	3.21	−1.69	Notch signaling transcription factor
Corneal Limbal Progenitor (cluster 9)				
KRT14	1.17	1.06	−2.04	Epithelial keratin
KRT15	−1.32	1.07	−1.95	Epithelial keratin
CXCL14	−1.16	−1.03	1.14	Chemokine receptor
CEBPD	1.30	1.33	1.36	bZIP transcription factor
S100A2	−1.19	−1.37	−2.25	Calcium binding protein
TXNIP	−1.98	−1.71	1.12	Protects against oxidative stress
TP63	1.22	1.40	−1.28	Transcription factor p53 family
Corneal Limbal Neural Crest Derived Progenitor (cluster 10)				
KRT14	1.17	1.06	−2.04	Epithelial keratin
CPVL	−2.30	−3.49	1.26	Carboxypeptidase vitellogenic like
PAX6	1.41	−1.38	−1.02	Transcription factor; regulates eye development
TP63	1.22	1.40	−1.28	Transcription factor p53 family
Corneal Limbal Quiescent Stem Cell (cluster 13)				
GPHA2	Not expressed			Glycoprotein Hormone Subunit A2
CASP14	Not expressed			Caspase 14
Stromal Cell Types				
Corneal Central Stromal Keratocyte (cluster 12)				
KERA	Not expressed			Keratocan
LUM	−2.41	−1.58	2.89	Lumican
Corneal Limbal Stromal Keratocyte (cluster 16)				
FBLN1	1.11	1.05	2.82	Limbal Stem Cell Niche
COL1A1	−2.25	−4.04	2.63	Collagen of Limbal Stromal Niche
COL1A2	−1.47	−2.17	2.64	Collagen of Limbal Stromal Niche
COL3A1	−1.67	−2.40	3.01	Collagen of Limbal Stromal Niche
OGN	−1.23	−1.59	−1.06	Osteoglycin
Corneal Limbal Fibroblast (cluster 8)				
FBLN1	1.11	1.05	2.82	Fibrillin 1 limbal stem cell niche marker
Corneal Stromal Stem Cell (cluster 3)				
KERA	Not expressed			Keratocan
ENG (CD105)	Not expressed			Endoglin
Other Cell Types				
Corneal Endothelial Cells (cluster 20)				
ACKR1	Not expressed			Chemokine receptor
CDH18	Not expressed			Cadherin
Fibroblastic Corneal Endothelial Cells (cluster 17)				
TAGLN	−1.22	−1.09	2.77	Calponin family actin binding protein transgelin
ACTA2	−1.39	−1.25	2.60	Smooth muscle actin
Endothelial Cells of Vessels (cluster 11)				
ACKR1	Not expressed			Chemokine receptor
POSTN	−7.13	−4.82	12.06	Matricellular protein periostin
Blood Cells (cluster 14)				
HBA1	−13.53	−14.70	13.58	
Lymphatic Vessels (cluster 18)				
ACKR1	Not expressed			Chemokine receptor
CCL21	−1.62	−1.14	2.99	Chemokine
LYVE1	1.19	−1.36	1.93	Lymphatic vessel endothelial hyaluronan receptor 1
Immune Cells I (cluster 15)				
CCL3	Not expressed			Chemokine
Immune Cells II (cluster 17)				
CCL5	−2.57	−2.56	2.98	Chemokine
Melanocytes (cluster 19)				
MLANA	−2.32	−1.55	1.88	Stabilizes PMEL
PMEL	−2.98	−1.39	1.69	Melanosome marker
MITF	−1.46	1.06	2.84	Melanocyte inducing transcription factor
TYRP1	−2.77	1.16	1.64	Tyrosinase family melanosomal enzyme
TYR	−2.60	−1.61	1.65	Tyrosinase family melanosomal enzyme
Color Key				
DEGs 5-fold higher than conjunctiva				DEGs 5-fold lower than conjunctiva
DEGs 4 to-5-fold higher than conjunctiva				DEGs 4 to-5-fold lower than conjunctiva
DEGs 3 to 4-fold higher than conjunctiva				DEGs 3-fold lower than conjunctiva
DEGs 2 to 3-fold higher than conjunctiva				DEGs 2 to 3-fold lower than conjunctiva
DEGs 1.5 to 2-fold higher than conjunctiva				DEGs 1.5 to 2-fold lower than conjunctiva

**Table 7:** Upstream Regulators Predicted by IPA.

Tissue Comparison	Upstream Regulator: Pathway Activated				Upstream Regulator: Pathway Inhibited			
	Epithelial Cell Proliferation	Epithelial Cell Fate	Inflammation	Stress Response	Epithelial- Mesenchymal Transition	DNA Damage Response	Immune Response	Fibrovascular Proliferation
Conjunctiva vs. Pinguecula								
Conjunctiva vs. Pterygium-E	PDGFBB, PRKCE, EGF, HGF, TNFA, ERK, MEK		IL1B, ECSIT				IgG	
Conjunctiva vs. Pterygium-NE	HGF, SRC	MYC					IgG, PTGER	
Pinguecula vs. Pterygium-E	PGFBB, ERBB2, ERK, CREB1	MYC, TP63, KLF4			SNAI1		IgG	VCAN
Pinguecula vs. Pterygium-NE	KRAS	MYC, TP63, KLF4		NUPRI1		FOXM1	IgG, CSF2, OSM	
Pterygium-E vs. Pterygium-NE								

## Data Availability

Raw sequencing data and metadata have been deposited in the Gene Expression Omnibus (GEO) data repository, of the National Center for Biotechnology Information, U.S. National Library of Medicine, Bethesda, MD, USA with accession numbers GSM5552151 to GSM5552158.

## Supplementary Materials

The following are available online at www.mdpi.com/article/10.3390/ijms222112090/s1. Table S1: Pathological Characteristics of Pterygium, Table S2: Functions of all Upregulated Gene Products on Top 25 Lists, Table S3: Functions of all Downregulated Gene Products on Top 25 Lists, Table S4: Changes in Expression of Cell Type Signature Genes in Mouse, Table S5: Epithelial Cell Proliferation, Table S6: Epithelial Cell Fate, Table S7: Notch and Wnt Signaling, Table S8: Keratin Genes Expressed, Table S9: Transient Receptor Potential (TRP) Channels Expressed, Table S10: Keratinization and Cornification, Table S11: Mucin Genes Expressed. Table S12: Angiogenesis, Table S13: Extracellular Matrix and Cell Surface Receptors, Table S14: MMPs and TIMPs, Table S15: Oligonucleotide Primers Used for qPCR [115,116,117,118,119,120,121,122,123].
