# Supplementary material for: Transcriptome Analysis of Pterygium and Pinguecula Reveals Evidence of Genomic Instability Associated with Chronic Inflammation
Source: Int J Mol Sci. 2021 Nov 8;22(21):12090. doi: 10.3390/ijms222112090 (PMC8584501; doi:10.3390/ijms222112090)
Supplement: Supplementary file 1 [file ijms-22-12090-s001.zip › ijms-1392887-supplementary.pdf]

Supplementary Materials

Table S1. Pathological Characteristics of Pterygium

Corresponding Author, Year Published, Citation	Di Girolamo 2011 [26]	Reda 2018 [27]	Bergeron 2021 [28]
Number and Type of Specimens	n=59, primary n=41, recurrent	n=47	n=149
Location of Study	Sydney, Australia	Cairo, Egypt	Montreal, Canada
EPITHELIAL			
Atypia, dysplasia or neoplasia	12%	53.2%	20.8%
Squamous metaplasia, cell proliferation, migratory front	Common	83%	62%
Epithelial atrophy			26%
Melanocyte hyperplasia	Common	48.9%	31.5%
Goblet cell hyperplasia	Prominent	39.1%	5%
Fuch's flecks: basal epithelial cell clusters, basal cell hyperplasia	18%		28%
STROMAL			
Bowman's membrane dissolution	Common		
Solar elastosis	Common	100%	35%
Stromal plaques (associated with solar elastosis)	6%		46%
Neovascularization		76.6%	54%
Neovascularization with Hemorrhage			32%
Stromal angiogenesis, hemorrhage, fibrovascular proliferation, ECM deposition	Common		
INFLAMMATORY			
Acute inflammation (neutrophils)			3%
Chronic inflammation (lymphoplasmacytic infiltrate)	60%	70 -75%	22%
Histiocytes			26%

Table S2. Functions of all Upregulated Gene Products on Top 25 Lists

Gene (HUGO Designation)	Function of Gene Product
Cell Cycle Checkpoint and Genomic Stability	
AATK	Induced during apoptosis. Its expression may be a necessary pre-requisite for the induction of growth arrest.
PLD6	Endonuclease thought to be involved in maintaining genomic stability.
SLC26A4-AS1	Inhibits expression of DNA double-strand breaks repair genes [115]
Epithelial Differentiation Marker	
KRT9	Keratin 9; specific to the palmoplantar epidermis
GJC2	Connexin gap junction protein
Mucosal Differentiation Marker	
MUC6	Secreted mucin; marker for gastric mucosal epithelia
Epithelial Cornification	
CAPN14	Epithelial cornification; distinguishes mucosal epithelium of the lung from epidermis.
FOSL1	Fos family transcription factor, dimerizes with JUN family members, forming stress response transcription factor complex AP-1.
SPRR3	Epithelial cornification
IVL	Epithelial cornification protein
Epithelial Cell Fate and Differentiation	
IGF1	Insulin Like Growth Factor 1; epithelial proliferation upstream regulator
NR2F1	Nuclear hormone receptor and transcriptional regulator; cancer dormancy gene [116].
PITX1	Transcription factor that expressed in epithelial progenitor cells. Downregulation associated with malignant transformation of mucosal epithelia. However, expression is higher in precancerous dysplastic epithelia [117].
POU5F1	Transcription factor; Expressed in the basal layer of the corneal epithelium [60].
PPM1N	Putative Mg2+/Mn2+ dependent Protein Phosphatase. The Gene Ontogeny Resource (GO) indicates that it is involved in negative regulation of I-kappaB kinase/NF-kappaB signaling and positive regulation of the canonical Wnt signaling pathway.
SNAI1	Regulation of epithelial-mesenchymal transition (EMT); also, involved in fibrovascular proliferation
Glycolysis and Gluconeogenesis	
LDHAL6B	Metabolic enzyme of the glycolysis/gluconeogenesis pathway
PCK1	Metabolic enzyme of the glycolysis/gluconeogenesis pathway
TMPRSS11B	promotes tumor growth by enhancing lactate export [31]
ALDH1	Alcohol dehydrogenase
ADH1C	Alcohol dehydrogenase
Protection against UV Light	
LGI3	UVB irradiation stimulates secretion of LGI3 protein, which protects against deleterious effects of UVB [35]
NPIPA3	Nuclear Pore Complex Interacting Protein; high expression in the retinal rod photoreceptors of the macula [36].
Expressed by Lymphocytes	
CCR2	Receptor for CCL2, a chemokine
CD2	Surface antigen found on all peripheral T-cells. Interacts with CD58 (LFA3) on antigen-presenting cells to optimize immune recognition.
IDO1	Indoleamine 2,3-dioxygenase; expressed in dendritic cells, monocytes, and macrophages, modulates T-cell behavior by its pericellular catabolization of the essential amino acid tryptophan
NR2F1	Orphan nuclear receptor and transcriptional regulator implicated in lymphocyte biology [118]
SFRP2	Wnt signaling modulator
Expressed by Lymphocytes: Immunoglobulin Chains	
IGHA1	Immunoglobulin Heavy Constant Alpha 1
IGHA2	Immunoglobulin Heavy Constant Alpha 2
IGHG1	Immunoglobulin Heavy Constant Gamma 1
IGHM	Immunoglobulin Heavy Constant Mu
IGKC	Immunoglobulin Heavy Kappa Constant
IGLC2	Immunoglobulin Lambda Constant 2
IGLC3	Immunoglobulin Lambda Constant 3
Immune Response	
CLEC18A	Lectin that functions as a co-receptor for TLR3 (toll-like receptor 3)
Fibrovascular Proliferation	
HBA1	Hemoglobin; red blood cell marker

PI16	Protease inhibitor produced by vascular endothelial cells.
POSTN	Periostin; ligand for alphaV/beta3 and alphaV/beta5 integrins; supports adhesion and migration of epithelial cells and vascular endothelial cells; [119].
RP1-261G23.7	Long noncoding RNA, antisense to VEGF, promotes VEGF gene expression [120].
Other	
AC004943.1	Transcription factor
CGB7	Beta subunit of chorionic gonadotropin. The alpha subunit is not transcribed in our samples.
DSPP	Dentin sialophosphoprotein
FAM71A	Rab2B interacting protein important in Golgi body integrity.
MYCT1	Overexpression mediates many of the known phenotypic features associated with transcription factor MYC.
PEAK3	(Probably) inactive protein kinase that acts as a scaffolding protein regulating cytoskeleton to control cell spreading and migration.
Unknown Function (11 genes)	
AC007000.12, LSMEM1, PALM2-AKAP2, RP5-1126H10.2, RP5-1142A6.8, RP11-164P12.3, RP11-556K13.1, RP11-78A19.3, RP11-848G14.5, TMEM254-AS1, Z95704.2	
Pseudogenes (7 genes)	
CCDC163P, EIF4HP2, FABP5P3, FAM25HP, FOSL1P, RNU2-59P, ZBTB45P1	

Table S3. Functions of all Downregulated Gene Products on Top 25 Lists

Gene (HUGO Designation)	Function of Gene Product
Tumor Suppressors	
C10orf90	Fragile Site Associated Tumor Suppressor; FATS; intracellular protein, promotes the activation of p53 in response to DNA damage, leading to a robust checkpoint response [37]. Chromosomal location at a common fragile site (CFS), susceptible to deletion in tumors induced by ionizing radiation.
DMBT1	Deleted in Malignant Brain Tumors 1; small secreted protein expressed at high levels by mucosal tissues [39,40].
RARRES1	Retinoic Acid Receptor Responder 1); a type I membrane protein. When *RARRES1* is depleted in epithelial cells, they rewire glucose metabolism by switching from aerobic glycolysis to glucose-dependent de novo lipogenesis [38].
SCGB3A1	Secretoglobin Family 3A Member 1; small secreted protein expressed at high levels by mucosal tissues [41].
Immune Response	
C3	Complement Component 3
CCL18	Chemokine that attracts T-cells into tissues
FCGR3A	Receptor for Fc-gamma, expressed on the surface of leukocytes (NK and neutrophils), that mediates antibody-dependent cellular cytotoxicity and other antibody-dependent responses
FAIM3	Fc Receptor for IgM
IGHG3	Immunoglobulin
MRC1	Mannose receptor of the lectin family; alternative pathway for complement activation
Molecular Chaperones	
HSPA1A	HSP70 family
HSPA1B	HSP70 family
HSPA6	HSP70 family
HSPA7	HSP70 family
Detoxifying Enzymes	
AKR1B10	Aldo-keto reductase detoxification enzyme
CYP1B1-AS1	Cytochrome P450 family detoxifying enzyme antisense RNA
Epithelial Cell Type Markers	
S100B	Epithelial cell type marker
Mucosal Markers	
MUC7	Secreted mucin
Angiogenesis	
HBA1	Hemoglobin subunit; red blood cell marker
HBA2	Hemoglobin subunit; red blood cell marker
HBB	Hemoglobin subunit; red blood cell marker
Other	
ATP6V1B1	Component of vacuolar ATPase that mediates acidification
POLR2J2	RNA polymerase II subunit
RNU1-2	Small nuclear RNA
RNU1-4	Small nuclear RNA
SNORA11D	Small nucleolar RNA
TAS2R46	G-protein-coupled taste receptor
WDR72	Promotes amylogenesis
ZSCAN23	zinc finger transcription factor
Unknown Functions (16 genes)	
AC084219.3, AC087392.1, CTD-3232M19.2, CTC-432M15.3, LINC00623, RP3-522J7.5, RP4-737E23.2, RP11-302B13.5, RP11-530C5.1, RP11-552F3.12, RP11-713P17.3, RP11-74C1.4, RP11-75A9.3, RP11-85F14.5, RPL36A-HNRNPH2, TSNAX-DISC1	
Pseudogenes (8 genes)	
ATP1B1P1, EEF1B2P1, HERC2P5, KRT18P60, PFN1P3, RPSAP41, USP32P2, ZNF322P1	

Table S4. Changes in Expression of Cell Type Signature Genes in Mouse

Gene (HUGO Designation)	Fold-change vs conjunctiva			Function
	Pterygium-E	Pterygium-NE	Pinguecula	
EPITHELIAL CELL TYPES				
Superficial Differentiated				
GJB4	1.68	1.21	-2.31	Connexin gap junction protein
Corneal				
PBK	-4.09	-5.07	-1.61	
MUC4	2.48	1.09	1.35	Corneal mucin
H2AX	Not expressed			Histone that is acetylated in DNA damage
Conjunctival				
KRT4	2.39	4.37	-1.20	
KRT13	1.57	3.34	-1.68	
MUC1	1.27	1.18	-1.06	
MUC20	1.50	1.27	-1.60	
Mature Transit Amplifying Cells				
MKI67	-1.70	-3.78	1.29	
Corneal Limbal Basal				
TXNIP	-1.98	-1.71	1.12	
Corneal Limbal Progenitor				
LRIG2	-1.39	-1.23	-1.27	
KRT19	1.28	1.37	1.03	Corneal limbal stem cells
STROMAL CELL TYPES				
Stromal Cells				
KERA	Not expressed			
LUM	-2.41	-1.58	2.89	
VIM	-1.64	-1.65	2.29	
Corneal Stromal Stem Cell				
KERA	Not expressed			
SCF	Not expressed			
FH1	Not expressed			
CD90	Not expressed			
SIX2	-1.14	-1.67	1.31	

Color Key

DEGs 5-fold higher than conjunctiva	DEGs 5-fold lower than conjunctiva
DEGs 4 to-5-fold higher than conjunctiva	DEGs 4 to-5-fold lower than conjunctiva
DEGs 3 to 4-fold higher than conjunctiva	DEGs 3 to 4-fold lower than conjunctiva
DEGs 2 to 3-fold higher than conjunctiva	DEGs 2 to 3-fold lower than conjunctiva
DEGs 1.5 to 2-fold higher than conjunctiva	DEGs 1.5 to 2-fold lower than conjunctiva

Table S5. Epithelial Cell Proliferation

Gene (HUGO Designation)	Fold-change vs conjunctiva			Protein Function
	Pterygium-E	Pterygium-NE	Pinguecula	
LIGANDS				
IGF1	-1.18	-2.07	16.78	Insulin-Like Growth Factor 1
TGFA	1.41	1.24	-1.33	Transforming Growth Factor Alpha
EGF	1.20	1.25	-1.70	Epidermal Growth Factor
HGF	1.05	-1.47	6.77	Hepatocyte Growth Factor
HBEGF	4.55	3.49	1.34	Heparin Bound Epidermal Growth Factor
AREG	1.77	5.03	1.40	Amphiregulin
AREGB	1.98	4.53	1.35	Amphiregulin B
EREG	1.87	1.75	1.23	Epiregulin
NRG1	1.14	1.62	-1.33	Neuregulin 1
NRG2	1.35	1.37	1.22	Neuregulin 2
NRG4	1.98	1.94	1.03	Neuregulin 4
CELL SURFACE RECEPTORS				
IGF1R	-1.05	-1.12	-1.01	IGF1 Receptor
EGFR	-1.08	1.21	-1.24	EGF Receptor
EGFR-AS	1.38	1.20	-1.36	EGF Receptor Antisense RNA
ERBB2	1.41	1.16	-1.03	
ERBB3	1.25	-1.03	1.04	
ERBB4	1.10	-1.01	1.28	
MET	-1.02	1.13	-1.33	
CO-RECEPTORS				
ITGAV	-1.48	-1.53	1.01	Integrin Alpha V
ITGB3	-1.87	-1.79	1.43	Integrin Beta 3
ITGA6	1.12	1.45	-1.09	Integrin Alpha 6
ITGB4	1.06	1.00	-1.39	Integrin Beta 4
SMALL GTPASES				
HRAS	1.27	1.43	-1.29	
KRAS	1.13	1.29	1.20	
NRAS	1.15	1.38	-1.13	
RASA1	-1.24	-1.28	1.28	
RASA2	1.20	1.02	1.06	
PROTEIN KINASES				
AKT1	1.02	1.01	-1.00	
AKT2	1.24	-1.20	-1.14	
AKT3	-1.93	-1.74	1.83	Protein Kinase B
MAPK1 (ERK)	1.04	-1.03	1.03	
MAP2K1P1 (MEK)	-2.04	-1.86	-1.08	
PIK3CA	-1.09	-1.33	1.21	
DISEASE-ASSOCIATED GENE				
PSORS1C1	4.64	2.22	1.65	Psoriasis Susceptibility 1 Candidate 1; unknown function

Color Key

DEGs 5-fold higher than conjunctiva	DEGs 5-fold lower than conjunctiva
DEGs 4 to-5-fold higher than conjunctiva	DEGs 4 to-5-fold lower than conjunctiva
DEGs 3 to 4-fold higher than conjunctiva	DEGs 3 to 4-fold lower than conjunctiva
DEGs 2 to 3-fold higher than conjunctiva	DEGs 2 to 3-fold lower than conjunctiva
DEGs 1.5 to 2-fold higher than conjunctiva	DEGs 1.5 to 2-fold lower than conjunctiva

Table S6. Epithelial Cell Fate

Gene (HUGO Designation)	Fold-change vs conjunctiva			Protein Function
	Pterygium-E	Pterygium-NE	Pinguecula	
TRANSCRIPTION FACTORS				
EHF	1.23	1.02	1.20	
KLF4	1.81	1.22	1.35	
KLF5	1.41	1.11	-1.12	
KLF7	-1.22	-1.12	1.37	
KLF10	2.15	2.49	1.33	Works through TGFB signaling
MYC	2.34	3.25	-1.37	
PAX6	1.41	-1.38	-1.02	Master regulator of eye development
PITX1	9.86	8.20	1.36	
POU5F1	9.36	9.09	1.84	
SNAI1	2.18	11.98	6.09	
TP63	1.22	1.40	-1.28	
SECRETED LIGANDS				
BMP1	1.40	1.18	-1.22	Bone Morphogenetic Protein 1
BMP2	1.65	2.22	-1.01	Bone Morphogenetic Protein 2
BMP3	1.33	-1.03	1.53	Bone Morphogenetic Protein 3
BMP4	1.43	1.24	1.38	Bone Morphogenetic Protein 4
BMP6	1.23	1.58	2.61	Bone Morphogenetic Protein 6
BMP7	1.49	1.43	-1.28	Bone Morphogenetic Protein 7
TGFB1	1.34	1.06	1.12	Transforming Growth Factor Beta 1
TGFB2	-1.20	-1.66	3.61	Transforming Growth Factor Beta 2
TGFB3	-1.43	-1.66	2.31	Transforming Growth Factor Beta 3

Color Key

DEGs 5-fold higher than conjunctiva	DEGs 5-fold lower than conjunctiva
DEGs 4 to-5-fold higher than conjunctiva	DEGs 4 to-5-fold lower than conjunctiva
DEGs 3 to 4-fold higher than conjunctiva	DEGs 3 to 4-fold lower than conjunctiva
DEGs 2 to 3-fold higher than conjunctiva	DEGs 2 to 3-fold lower than conjunctiva
DEGs 1.5 to 2-fold higher than conjunctiva	DEGs 1.5 to 2-fold lower than conjunctiva

Table S7. Notch and Wnt Signaling

Gene	Fold-change vs conjunctiva		
	Pterygium-E	Pterygium-NE	Pinguecula
NOTCH SIGNALING			
LIGANDS			
DLL1	-1.02	1.01	1.08
DLL4	-1.76	1.08	1.30
JAG1	1.38	1.57	1.17
JAG2	-1.22	-1.10	-1.13
RECEPTORS			
NOTCH1	1.39	1.22	-1.30
NOTCH2	1.25	1.16	1.15
NOTCH3	1.60	1.20	-1.14
NOTCH4	-3.22	-1.54	1.37
ENDOCYTIC ADAPTORS			
NUMB	1.09	-1.20	1.16
NUMBL	3.00	2.67	1.02
TRANSCRIPTION FACTORS			
HES1	1.04	-1.32	-1.13
HES2	1.26	1.40	-1.72
HES4	1.46	1.45	-1.40
HES5	6.02	3.21	-1.69
HES6	-2.47	-1.46	-1.74
TRANSCRIPTIONAL COACTIVATORS			
MAML1	1.03	-1.06	-1.12
MAML2	-1.05	-1.03	-1.11
MAML3	1.32	1.03	1.03
MAMLD1	-1.49	-1.74	1.11
WNT SIGNALING			
CATENINS			
CTNNB1	-1.17	-1.15	1.21
TUMOR SUPRESSORS			
APC	1.02	-1.11	1.27
AXIN1	1.51	1.34	-1.20
AXIN2	1.05	1.30	2.37
PROTEIN KINASES			
CSNK1A1	1.20	1.34	-1.03
GSK3A	1.50	1.34	1.12
PROTEIN PHOSPHATASES			
PPM1N	5.87	17.06	9.54
LIGANDS			
WNT2B	1.63	1.28	1.62
WNT3	-1.36	-1.54	1.08
WNT4	1.24	-1.02	-1.11
WNT3A	1.44	-1.07	1.05
WNT5A	1.04	-1.11	1.37
WNT5B	1.02	-1.58	2.00
WNT7A	1.09	1.05	-1.35
WNT7B	2.29	2.14	-1.19
WNT9A	3.76	3.50	-1.11
WNT10A	-1.71	-1.20	-1.41
INHIBITORS			
DKK2	-1.60	-1.68	3.48
DKK3	-1.46	-1.76	-1.10
DKK4	-1.74	-1.20	-1.22
SFRP1	1.33	2.46	1.55
SFRP2	2.38	1.43	14.57
FRZB	-2.13	-1.63	2.08
SFRP4	1.41	-2.79	6.09
RECEPTORS			
FZD1	-1.12	-1.15	1.47
FZD3	-1.22	-1.29	1.35
FZD4	-1.78	-1.38	3.75
FZD5	-1.01	-1.12	1.04
FZD6	-1.48	-1.63	-1.07
FZD7	1.00	1.21	1.32
FZD8	2.00	1.43	-1.35
FZD10	1.43	2.23	1.06
FZD10-AS1	1.11	3.98	1.74
RECEPTOR INTERACTING PROTEINS			
DVL1	2.07	1.47	-1.07
DVL2	1.10	1.08	-1.36
DVL3	1.34	1.04	1.06

OTHER ACTIVATING PROTEINS			
LGR4	1.00	-1.08	1.06
LGR5	2.63	-1.55	12.58
TRANSCRIPTION FACTORS			
TCF4	-1.67	-1.37	2.11
TCF7	2.12	2.39	2.08
Color Key			
DEGs 5-fold higher than conjunctiva			
DEGs 4 to-5-fold higher than conjunctiva			
DEGs 3 to 4-fold higher than conjunctiva			
DEGs 2 to 3-fold higher than conjunctiva			
DEGs 1.5 to 2-fold higher than conjunctiva			
DEGs 5-fold lower than conjunctiva			
DEGs 4 to-5-fold lower than conjunctiva			
DEGs 3 to 4-fold lower than conjunctiva			
DEGs 2 to 3-fold lower than conjunctiva			
DEGs 1.5 to 2-fold lower than conjunctiva			

Table S8. Keratin Genes Expressed

Gene (HUGO Designation)		Fold-change vs conjunctiva			Known expressing epithelial cell type					
Type I acidic KRT9-19	Type II basic KRT1-8	Pterygium-E	Pterygium-NE	Pinguecula	Cornea	Conj.	Limbal	Goblet	MBG	Skin
Keratins of corneal-type epithelia (basal and suprabasal)										
	KRT3	2.88	2.13	-1.53	X		X			
KRT12		1.28	-1.67	-1.84	X		X			
Keratins of mucosal and other stratifying soft epithelia (suprabasal)										
	KRT4	2.39	4.37	-1.20	X	X				
KRT10		1.56	1.62	-1.24	X	X	X		X	X
KRT13		1.57	3.34	-1.68	X	X				
KRT78		2.63	5.87	-1.46	Tongue					
KRT80		1.85	3.99	1.14	Tongue					
Keratins of stratifying epithelia (basal)										
	KRT5	1.64	1.89	-1.64	X	X	X			X
KRT14		1.17	1.06	-2.04	X	X	X			X
KRT15		-1.32	1.07	-1.95	X	X	X			X
Keratins of hyperproliferative epithelia (basal)										
	KRT6A	1.64	1.43	-1.48	X	X	X			X
	KRT6B	3.54	1.00	-1.36	X	X	X			X
	KRT6C	3.68	2.12	-1.09	X	X	X			X
KRT16		4.48	2.65	-1.41	X	X				X
KRT17		3.40	6.38	-1.68	X	X	X			X
Keratins of hyperproliferative epithelia (suprabasal)										
KRT9		8.25	4.74	11.86	Palmoplantar epidermis					
Keratins of simple epithelia										
	KRT7	1.37	1.35	-1.17		X		X		
	KRT8	1.27	-1.03	-1.83	X	X	X			X
KRT18		1.49	1.10	-1.69	X	X	X			X
KRT19		1.28	1.37	1.03	X	X	X			X
KRT23		1.40	1.51	1.25	Not previously identified in ocular surface epithelia					
KRT24		-3.34	2.63	-1.45			X			
Keratins of hard epithelia										
KRT31		-2.51	-1.46	-1.92	Hair cortex, nail matrix					
KRT40		1.30	1.53	1.31	Upper hair cuticle					
Unknown										
KRT222		-1.60	-1.33	1.65						

Color Key

DEGs 5-fold higher than conjunctiva	DEGs 5-fold lower than conjunctiva
DEGs 4 to-5-fold higher than conjunctiva	DEGs 4 to-5-fold lower than conjunctiva
DEGs 3 to 4-fold higher than conjunctiva	DEGs 3 to 4-fold lower than conjunctiva
DEGs 2 to 3-fold higher than conjunctiva	DEGs 2 to 3-fold lower than conjunctiva
DEGs 1.5 to 2-fold higher than conjunctiva	DEGs 1.5 to 2-fold lower than conjunctiva

Abbreviations:

Conj.: conjunctiva; Goblet: conjunctival goblet cell; MBG: Meibomian gland

Subcategory Definitions:

- Corneal-type epithelial keratins (suprabasal): expression specific to the corneal epithelium and limbal progenitors.
- Mucosal and other soft epithelial keratins (suprabasal): keratins expressed suprabasally in the conjunctival epithelium belong to this group. Keratin 10 is a Meibomian gland marker in mice [17].
- Stratifying epithelial keratins (basal): Expressed in basal cells and progenitor cells of stratifying epithelial tissues.
- Hyperproliferative epithelial keratins (basal): expressed in stratified squamous epithelia including the ocular surface epithelia, the epidermis, the tongue, the hair follicles and the palmoplantar epidermis; upregulated in healing skin wounds and hyperproliferative epidermal disorders [18], and in conjunctival squamous cell carcinoma [19].
- Simple epithelial keratins: expressed in simple (single-layered) epithelia. Keratin 7 is a goblet cell marker [20].
- Hard epithelial keratins: Expressed in hair and nails.

Table S9. Transient Receptor Potential (TRP) Channels Expressed

Gene (HUGO Designation)	Fold-change vs conjunctiva			Function
	Pterygium-E	Pterygium-NE	Pinguecula	
TRPC1	**-1.60**	**-1.62**	**1.56**	Transient receptor potential cation channel subfamily C member 1
TRPC6	**-2.48**	**-1.63**	**1.83**	Transient receptor potential cation channel subfamily C member 6
TRPM1	**-2.94**	**-1.65**	**1.01**	Transient receptor potential cation channel subfamily M member 1
TRPM2	**-2.05**	**-1.89**	**-1.13**	Transient receptor potential cation channel subfamily M member 2
TRPM3	**1.69**	**-1.33**	**1.41**	Transient receptor potential cation channel subfamily M member 3
TRPM4	**1.57**	**1.72**	**1.09**	Transient receptor potential cation channel subfamily M member 4
TRPM6	**-1.78**	**-1.90**	**-1.34**	Transient receptor potential cation channel subfamily M member 6
TRPM7	**-1.35**	**-1.30**	**1.14**	Transient receptor potential cation channel subfamily M member 7
TRPV1	**1.56**	**-1.03**	**1.07**	Transient receptor potential cation channel subfamily V member 1
TRPV2	**-1.19**	**-1.15**	**5.07**	Transient receptor potential cation channel subfamily V member 2
TRPV3	**5.57**	**6.23**	**1.53**	Transient receptor potential cation channel subfamily V member 3
TRPV4	**1.39**	**-1.07**	**1.41**	Transient receptor potential cation channel subfamily V member 4
TRPV6	**-1.23**	**-1.53**	**1.02**	Transient receptor potential cation channel subfamily V member 5

Color Key

DEGs 5-fold higher than conjunctiva	DEGs 5-fold lower than conjunctiva
DEGs 4 to-5-fold higher than conjunctiva	DEGs 4 to-5-fold lower than conjunctiva
DEGs 3 to 4-fold higher than conjunctiva	DEGs 3 to 4-fold lower than conjunctiva
DEGs 2 to 3-fold higher than conjunctiva	DEGs 2 to 3-fold lower than conjunctiva
DEGs 1.5 to 2-fold higher than conjunctiva	DEGs 1.5 to 2-fold lower than conjunctiva

Table S10. Keratinization and Cornification

Gene (HUGO Designation)	Fold-change vs conjunctiva			Protein Function
	Pterygium-E	Pterygium-NE	Pinguecula	
Regulatory Transcription Factor				
SNAI1	2.18	11.98	6.09	transcriptional repressor; epithelial-mesenchymal transition
FOSL1	2.97	14.79	-1.26	
PAX6	1.41	-1.38	-1.02	Master regulator of eye development
Conjunctival Differentiation Markers				
KRT4	2.39	4.37	-1.20	Conjunctival keratin
KRT14	1.57	3.34	-1.68	Conjunctival keratin
Keratin Aggregation				
KRT10	1.56	1.62	-1.24	Epidermal keratin
FLG2	-1.09	1.26	1.65	Filaggrin 2
FLG	-1.19	-1.38	1.20	Filaggrin
KRT1	Not expressed			Epidermal keratin
Desmosome Assembly				
PPL	2.03	2.58	-1.30	Periplakin
PKP1	2.41	2.35	-1.37	Plakophilin 1
DSP	1.57	1.97	-1.25	Desmoplakin
EVPL	2.27	1.93	-1.34	Envoplakin
DSG3	1.15	1.92	-1.34	Desmoglein 3
PERP	-1.09	1.06	-1.50	P53 Apoptosis Effector Related to PMP22
Cornified Envelope Assembly				
SPRR3	2.75	34.38	1.48	Small Proline Rich Protein 3
IVL	3.83	28.63	-1.27	Involucrin
CAPN14	4.27	10.73	-2.37	Calpain 14
SPRR1B	2.57	3.39	-1.65	Small Proline Rich Protein 1B
TGM4	4.30	2.65	1.04	Transglutaminase 4
SPRR2A	1.65	2.50	-1.66	Small Proline Rich Protein 2A
ABCA12	1.10	2.20	-1.63	Transporter involved in lipid homeostasis
CSTA	-1.30	2.05	-1.27	Cystatin A; precursor to cornified envelope
SCEL	1.27	2.14	-1.12	
TCHH	-1.29	1.07	-1.11	Trichohyalin
Desquamation				
KLK7	3.16	8.84	5.08	Kallikrein Related Peptidase 7
IL1RN	1.91	4.49	-1.76	Interleukin 1 receptor antagonist
SPINK5	-1.10	2.47	-1.69	Serine Peptidase Inhibitor Kazal Type 5
KAZN	1.69	1.64	-1.19	Kazrin; inhibits KLK5

Color Key

DEGs 5-fold higher than conjunctiva	DEGs 5-fold lower than conjunctiva
DEGs 4 to-5-fold higher than conjunctiva	DEGs 4 to-5-fold lower than conjunctiva
DEGs 3 to 4-fold higher than conjunctiva	DEGs 3 to 4-fold lower than conjunctiva
DEGs 2 to 3-fold higher than conjunctiva	DEGs 2 to 3-fold lower than conjunctiva
DEGs 1.5 to 2-fold higher than conjunctiva	DEGs 1.5 to 2-fold lower than conjunctiva

Table S11. Mucin Genes Expressed

Gene (HUGO Designation)	Fold-change vs conjunctiva			Function
	Pterygium-E	Pterygium-NE	Pinguecula	
Secreted mucins				
MUC2	-2.28	-7.22	-1.54	Gel-forming
MUC5AC	-1.76	-1.78	3.31	Gel-forming
MUC5B	-1.82	1.10	1.42	Gel-forming
MUC6	8.45	10.96	2.62	Gel-forming
MUC7	-51.82	-8.48	-2.36	Soluble monomer
OVGP1	1.49	1.52	-1.43	Soluble monomer (previously known as MUC9)
MUC19	Not expressed			
Membrane-associated mucins				
MUC1	1.27	1.18	-1.06	
MUC3A	-2.67	-2.11	-2.30	
MUC4	2.48	1.09	1.35	
MUC12	-1.42	-1.30	-1.29	
MUC13	Not expressed			
MUC15	1.17	1.22	-1.03	
MUC16	2.28	1.22	1.01	
MUC17	Not expressed			
MUC20	1.50	1.27	-1.60	
MUC21	1.60	1.95	-2.09	
MUC22	1.08	1.60	-2.28	

Color Key

DEGs 5-fold higher than conjunctiva	DEGs 5-fold lower than conjunctiva
DEGs 4 to-5-fold higher than conjunctiva	DEGs 4 to-5-fold lower than conjunctiva
DEGs 3 to 4-fold higher than conjunctiva	DEGs 3- to 4fold lower than conjunctiva
DEGs 2 to 3-fold higher than conjunctiva	DEGs 2 to 3-fold lower than conjunctiva
DEGs 1.5 to 2-fold higher than conjunctiva	DEGs 1.5 to 2-fold lower than conjunctiva

Table S12. Angiogenesis

Gene (HUGO Designation)	Fold-change vs conjunctiva			
	Pterygium-E	Pterygium-NE	Pinguecula	
Markers of blood vessels				
HBA1	-13.53	-14.70	13.58	Hemoglobin Subunit Alpha 1
HBA2	-10.64	-10.17	3.76	Hemoglobin Subunit Alpha 2
HBB	-11.00	-11.87	5.39	Hemoglobin Subunit Beta
PI16	1.38	-1.09	26.83	Protease inhibitor that regulates vascular permeability
Angiogenesis regulators				
TYMP	-1.27	-2.61	-1.19	Promotes angiogenesis
VEGFA	4.26	2.15	-1.04	Promotes angiogenesis
RP1-261G23.7	13.43	9.05	1.22	VEGFA antisense; enhances VEGFA gene expression
RP11-203J24.8	9.66	9.95	-1.36	BAIAP2 antisense (barcode angiogenesis inhibitor-binding protein)

Color Key

DEGs 5-fold higher than conjunctiva	DEGs 5-fold lower than conjunctiva
DEGs 4 to-5-fold higher than conjunctiva	DEGs 4 to-5-fold lower than conjunctiva
DEGs 3 to 4-fold higher than conjunctiva	DEGs 3-fold lower than conjunctiva
DEGs 2 to 3-fold higher than conjunctiva	DEGs 2 to 3-fold lower than conjunctiva
DEGs 1.5 to 2-fold higher than conjunctiva	DEGs 1.5 to 2-fold lower than conjunctiva

Table S13. Extracellular Matrix and Cell Surface Receptors

Gene (HUGO Designation)	Fold-change vs conjunctiva			Function
	Pterygium-E	Pterygium-NE	Pinguecula	
Integrins				
ITGA1	-1.41	-1.19	6.42	Integrin Alpha 1; dimerizes with ITGB1; collagen/laminin receptor
ITGA2	1.06	1.06	-1.37	Integrin Alpha 2
ITGA3	1.56	1.13	-1.74	Integrin Alpha 3
ITGA4	-2.10	-3.80	4.31	Integrin Alpha 4; dimerizes with ITGB1 or ITGB7; fibronectin receptor
ITGA6	1.12	1.45	-1.09	Integrin Alpha 6
ITGAE	-1.90	-2.19	1.27	Integrin Alpha E
ITGAL	-2.00	-3.04	4.93	Integrin Alpha L; lymphocytes; dimerizes with ITGB3; binds ICAMs
ITGAM	-1.92	-3.13	-1.03	Integrin Alpha M
ITGAV	-1.48	-1.53	1.01	Integrin Alpha V
ITGB1	-1.09	-1.01	-1.11	Integrin Beta 1
ITGB3	-1.87	-1.79	1.43	Integrin Beta 3
ITGB4	1.06	1.00	-1.39	Integrin Beta 4
ITGB5	1.11	-1.01	-1.21	Integrin Beta 5
ITGB7	1.55	1.25	1.59	Integrin Beta 7
ITGB8	1.20	1.59	-1.13	Integrin Beta 8
Basement Membrane Zone Extracellular Matrix				
COL4A1	-1.82	-1.71	2.52	Collagen Type IV Alpha 1 Chain
COL4A2	-1.60	-1.24	2.01	Collagen Type IV Alpha 2 Chain
COL4A3	-1.61	-1.39	1.25	Collagen Type IV Alpha 3 Chain
COL4A4	-1.38	-1.29	1.00	Collagen Type IV Alpha 4 Chain
COL4A5	-1.13	-1.50	-1.17	Collagen Type IV Alpha 5 Chain
COL4A6	-1.37	-2.05	-1.96	Collagen Type IV Alpha 6 Chain
LAMA1	-1.84	-1.00	-4.22	Laminin Alpha 1
LAMA2	-1.76	-1.00	1.23	Laminin Alpha 2
LAMA3	-1.11	1.20	-1.28	Laminin Alpha 3
LAMA4	-1.41	-1.43	3.10	Laminin Alpha 4
LAMA5	1.61	1.11	-1.15	Laminin Alpha 5
FBLN1	1.11	1.05	2.82	Fibulin 1
FBLN2	1.14	-1.11	-1.08	Fibulin 2
FBLN5	-1.20	-1.17	3.46	Fibulin 5
FBLN7	-1.81	1.14	-1.33	Fibulin 7
FN1	-4.02	-4.85	3.17	Fibronectin 1
POSTN	-7.13	-4.82	12.06	Periostin
Basement Membrane Zone Extracellular Matrix				
ADAMTS14	5.41	3.15	-1.51	Type I procollagen N-propeptidase
DCN	-1.25	-1.27	1.99	Decorin
COL1A1	-2.25	-4.04	2.63	Collagen Type I Alpha Chain 1
COL1A2	-1.47	-2.17	2.64	Collagen Type I Alpha Chain 2
COL3A1	-1.67	-2.40	3.01	Collagen Type 3 Alpha Chain 1
COL5A1	-1.85	-2.39	1.83	Collagen Type 5 Alpha Chain 1
COL5A2	-2.68	-2.34	1.29	Collagen Type 5 Alpha Chain 2
COL5A3	1.12	1.28	-1.35	Collagen Type 5 Alpha Chain 3
COL6A1	-1.51	-1.62	1.59	Collagen Type 6 Alpha Chain 1
COL6A2	-1.03	-1.12	1.58	Collagen Type 6 Alpha Chain 2
COL6A3	-1.86	-2.58	1.87	Collagen Type 6 Alpha Chain 3
COL6A6	1.01	-3.11	-1.20	Collagen Type 6 Alpha Chain 6
COL7A1	-1.46	-1.80	-1.54	Collagen Type 7 Alpha Chain 1
COL8A1	-1.60	-1.19	4.24	Collagen Type 8 Alpha Chain 1
COL8A2	-1.70	-2.04	2.11	Collagen Type 8 Alpha Chain 2
COL9A2	-1.27	-1.21	-1.52	Collagen Type 9 Alpha Chain 2
COL10A1	-3.35	-1.94	-1.25	Collagen Type I0 Alpha Chain 1
COL11A2	-2.00	-1.39	1.55	Collagen Type I1 Alpha Chain 2
COL12A1	-2.37	-1.97	-1.07	Collagen Type I2 Alpha Chain 1
COL14A1	1.32	-1.60	4.47	Collagen Type I4 Alpha Chain 1
COL15A1	-1.36	-1.21	2.84	Collagen Type I5 Alpha Chain 1
COL16A1	-1.65	-1.39	-1.62	Collagen Type I6 Alpha Chain 1
COL17A1	-1.12	-1.45	-1.50	Collagen Type I7 Alpha Chain 1
COL18A1	-1.44	-1.51	-1.17	Collagen Type I8 Alpha Chain 1
COL21A1	1.57	-1.28	1.91	Collagen Type 2I Alpha Chain 1
COL23A1	1.13	1.14	1.39	Collagen Type 23 Alpha Chain 1
COL25A1	-1.23	1.20	2.02	Collagen Type 25 Alpha Chain 1
COL27A1	-2.39	-3.04	1.19	Collagen Type 27 Alpha Chain 1
COL28A1	-1.03	-1.74	1.22	Collagen Type 28 Alpha Chain 1
SPARC	-2.09	-1.75	1.84	SPARC

Color Key

DEGs 5-fold higher than conjunctiva	DEGs 5-fold lower than conjunctiva
DEGs 4 to-5-fold higher than conjunctiva	DEGs 4 to-5-fold lower than conjunctiva
DEGs 3 to 4-fold higher than conjunctiva	DEGs 3 to 4-fold lower than conjunctiva
DEGs 2 to 3-fold higher than conjunctiva	DEGs 2 to 3-fold lower than conjunctiva
DEGs 1.5 to 2-fold higher than conjunctiva	DEGs 1.5 to 2-fold lower than conjunctiva

Table S14. MMPs and TIMPs

Gene (HUGO Designation)	Fold-change vs conjunctiva			MMP genes upregulated in repairing mouse ocular surface epithelium [21]	
	Pterygium-E	Pterygium-NE	Pinguecula	Unwounded	Repairing
MMP1	Not expressed				
MMP1a	Mouse only				X
MMP1b	Mouse only				X
MMP2	-1.49	-2.47	2.07	X	
MMP3	-1.21	1.97	-1.83	X	
MMP7	-8.19	-9.18	-1.70	X	
MMP8	Not detected				
MMP9	Not detected				X
MMP10	1.19	-3.90	2.95		X
MMP11	Not detected				
MMP12	-1.33	-1.30	1.49		X
MMP13	Not detected				X
MMP14	1.19	-1.01	-1.05	X	
MMP15	1.30	-1.17	-1.78		
MMP16	-1.24	-1.52	3.30		
MMP17	-3.58	-1.76	1.25		
MMP19	-1.86	-1.43	-1.09		
MMP20	Not detected				
MMP21	Not detected				
MMP24	-1.28	-1.35	-1.99		
MMP24-AS1	1.21	-1.06	1.04		
MMP25	-5.08	-3.33	1.16		
MMP26	Not detected				
MMP28					
TIMP1	-1.18	-1.47	1.45		
TIMP2	-1.23	-1.42	1.77		
TIMP3	-1.43	-1.08	1.59		
TIMP4	Not detected				

Color Key

DEGs ≥ 5-fold higher than conjunctiva	DEGs ≥ 5-fold lower than conjunctiva
DEGs ≥ 4 to-5-fold higher than conjunctiva	DEGs ≥ 4 to-5-fold lower than conjunctiva
DEGs ≥ 3 to 4-fold higher than conjunctiva	DEGs ≥ 3-fold lower than conjunctiva
DEGs ≥ 2 to 3-fold higher than conjunctiva	DEGs ≥ 2 to 3-fold lower than conjunctiva
DEGs ≥ 1.5 to 2-fold higher than conjunctiva	DEGs ≥ 1.5 to 2-fold lower than conjunctiva

Table S15. Oligonucleotide Primers used for qPCR

**Gene**	**Forward Sequence**	**Reverse Sequence**
AATK	ccgatttccttcaggtacagg	tgtacgtcctgccactca
ATF4	tcaaacctcatgggttctcc	gtgtcatccaacgtggtcag
CHOP	agaaccaggaaacggaaacaga	tctccttcatgcgctgcttt
COL1A1	gaacgcgtgtcatcccttgt	gaacgaggtagtctttcagcaaca
DMBT1	tgtgaggaagttggaatagtcg	ccagataacgccgaacct
FN1	agcggacctacctaggcaat	ggtttgcgatggtacagctt
MUC7	atgccttctgtgacgtagttc	atgaaaactctgccgctgt
PITX1	cgtagccacccttgcac	ctaccccgacatgagcat
POU5F1	tgtgtctatctactgtgtccca	gttggagggaaggtgaagttc
PTN	ctcctgtttcttgccttcct	actgtcaccatctccaagc
SCGB3A1	gcagcgtcttgtcctcag	ctcatagagggctcccagaa
SIX1	accggaggcaaagagacc	ggagagagttggttctgcttgt
XBP1	ctgagtccgaatcaggtgcag	atccatggggagatgttctgg
GAPDH	ggatgatgttctggagagcc	catcaccatcttccaggagc
